# A Review on Electroactive Polymer–Metal Composites: Development and Applications for Tissue Regeneration

**DOI:** 10.3390/jfb14100523

**Published:** 2023-10-17

**Authors:** Rumi Acharya, Sayan Deb Dutta, Tejal V. Patil, Keya Ganguly, Aayushi Randhawa, Ki-Taek Lim

**Affiliations:** 1 Department of Biosystems Engineering, Kangwon National University, Chuncheon 24341, Republic of Korea; 2 Interdisciplinary Program in Smart Agriculture, Kangwon National University, Chuncheon 24341, Republic of Korea; 3 Institute of Forest Science, Kangwon National University, Chuncheon 24341, Republic of Korea

**Keywords:** electroactive polymer, biocompatibility, polymer–metal composites, tissue engineering

## Abstract

Electroactive polymer–metal composites (EAPMCs) have gained significant attention in tissue engineering owing to their exceptional mechanical and electrical properties. EAPMCs develop by combining an electroactive polymer matrix and a conductive metal. The design considerations include choosing an appropriate metal that provides mechanical strength and electrical conductivity and selecting an electroactive polymer that displays biocompatibility and electrical responsiveness. Interface engineering and surface modification techniques are also crucial for enhancing the adhesion and biocompatibility of composites. The potential of EAPMC-based tissue engineering revolves around its ability to promote cellular responses, such as cell adhesion, proliferation, and differentiation, through electrical stimulation. The electrical properties of these composites can be used to mimic natural electrical signals within tissues and organs, thereby aiding tissue regeneration. Furthermore, the mechanical characteristics of the metallic components provide structural reinforcement and can be modified to align with the distinct demands of various tissues. EAPMCs have extraordinary potential as regenerative biomaterials owing to their ability to promote beneficial effects in numerous electrically responsive cells. This study emphasizes the characteristics and applications of EAPMCs in tissue engineering.

## 1. Introduction

Electroactive polymers (EAPs) are flexible polymers that acquire mechanical strain in response to electrical stimuli, or produce voltage when mechanically deformed. Their characteristics have prompted researchers to consider their potential use as sensor actuators in biomimetic and standard robotics applications [1]. EAPs change their volume or form when subjected to an electrical field [2]. EAPs have mechanical responses to electrical stimulation. “Actuation” describes a system’s responsiveness to external stimuli, such as movement or the introduction of a new substance. Depending on the application, EAPs can be precisely controlled by altering their chemical and physical characteristics (i.e., biosensors, molecular targeting, and bioinstructive scaffolds) [3]. Conductive polymers (CPs) with conjugated pi-bond systems on their backbones generate an extensive chain of electrons that are only loosely bound. Ionic functional groups, such as carboxylic, sulfonic, phosphoric, and amine groups, can be introduced to any polymeric biomaterial to produce ionically conductive (IC) polymers [4] The cutting-edge science of tissue engineering in regenerative medicine creates viable biological alternatives for the repair, replacement, or augmentation of damaged tissues and organs. This discipline involves designing biomimetic constructions that interact with biological systems, and includes the fields of biology, chemistry, materials science, and engineering. Using EAP–metal composites (EAPMCs) to construct dynamic and responsive tissue scaffolds and implants is a potential approach to tissue engineering [1]. EAPMCs are innovative materials comprising EAPs and metals. Electrical stimulation affects the size, shape, and mechanical characteristics of EAPs, such as polypyrrole (PPy) and polythiophene (PTh) [5]. When paired with shape-memory alloys (SMAs) or conductive metals, EAPMCs can respond to electrical stimuli in highly regulated and predictable ways, which makes them interesting for tissue engineering, because dynamic and sensitive materials may better simulate biological tissues [6].

The integration of EAPMCs into tissue engineering has several advantages. First, their electrical signaling abilities can enhance cell adhesion, proliferation, and differentiation, thereby enabling tissue regeneration. Second, their mechanical characteristics can be adjusted to those of native tissue for maximum support and usefulness. EAPMCs can also be used to develop biohybrid systems that allow electrical devices and biological tissues to communicate, thereby enabling enhanced biomedical applications [7].

Various EAPs, such as conducting polymers, ferroelectric polymers, elastomers, and ionic polymers, are frequently employed [1]. This review article focuses on ionic polymer–metal composites and their potential applications in tissue engineering. Recent research on EAPs has shown their potential in the development of versatile soft robots [8]. These soft robots with compliant bodies and flexible actuators have gained significant attention in various fields, including biomedical applications [9]. One of the critical advantages of EAPMCs in tissue engineering is their high deformability and flexibility, which allows them to mimic the mechanical properties of biological tissues. Furthermore, EAPMCs have low operation voltages and significant strain capabilities, making them suitable for various tissue-engineering applications [10]. The process of electrical coupling and the total function of EAPMCs depends to a large degree on how the polymer matrix is determined. Therefore, choosing polymer materials to optimize the performance of EAPMC composites in tissue-engineering applications is very important [11]. The use of EAPMCs in tissue engineering offers several advantages [12]. Firstly, these composites can mimic the mechanical properties of natural tissues, which is crucial for tissue-engineering applications, as it allows for the development of biomimetic scaffolds that can provide mechanical support and promote cell attachment and growth [13]. Secondly, EAPMCs can undergo large deformations in response to small applied voltages. This property is fundamental in tissue engineering, as it allows for generating mechanical stimuli that can promote cell differentiation and tissue formation [14]. EAPMCs are well-suited for applications in biological systems, since their low operating voltages make them safe for cells and tissues. In addition, EAPMCs have been shown to have excellent biocompatibility, making them suitable for biomedical applications [15].

**Scheme 1:**
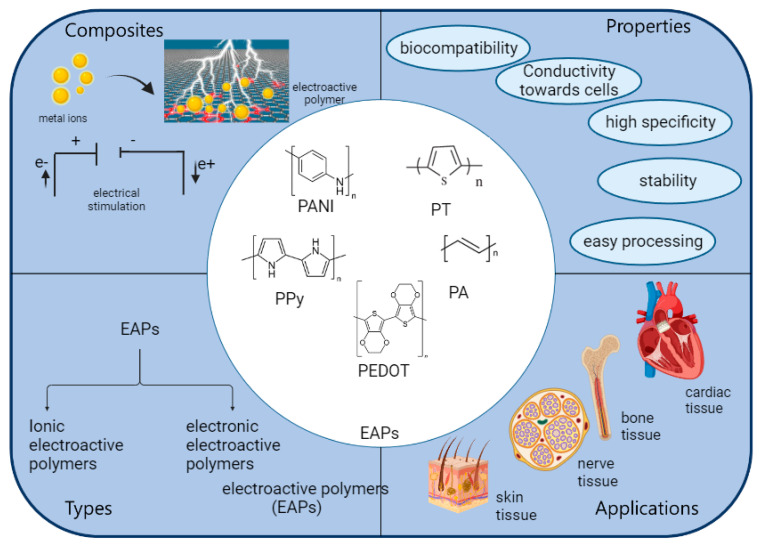
Representation of fabrication of EAPMC and properties for various tissue-engineering applications.

As shown in Scheme 1, this review article aims to provide an overview of current research and development on EAPMCs in tissue engineering. It highlights the attractive attributes of EAPs for electromechanical applications, specifically in actuators and sensors.

## 2. Effect of Electrical Stimulation on Biological Tissues

Recently, electrical stimulation (ES) has attracted considerable attention as a physical stimulus. Owing to its solid features, it exhibits considerable promise in the treatment of diseases and wound healing [16]. Cell proliferation, migration, and differentiation can all be affected by electrical stimulation, because it can activate various intracellular signaling pathways and alter the intracellular milieu [17].

Tissue engineering has recently become essential for researchers in health-care organizations, as it provides a novel and effective method for treating multiple injuries, burns, skin regeneration and bone fractures. For these purposes, tissue engineering is an alternative healing method, and tissue regeneration primarily aims to produce a scaffold appropriate to support cell growth [18]. Therefore, stimulation and handling need to be performed according to the targeted tissue type [19]. The utilized scaffold should include mechanical elements suitable for the force in the pertinent tissue and should be a biodegradable and nonimmunogenic visceral tissue, a type of natural-tissue-derived cell, definite progenitor cells, or stem cells [20]. Tissue-engineered systems have been differentiated and integrated into the target site using a variety of stimuli. ES and mechanical stimulation (MS) are the two external inputs currently being studied. By encouraging the release of bimolecular signals and growth factors, these external stimulation methods help cells to differentiate [21].

Based on how nerve cells react to an electric field, ES is an acknowledged method for controlling how these cells behave in vitro [22]. From this standpoint, it is crucial to understand how electrochemical stimulation can be adjusted to create an appropriate cell response. Most biological studies recommend applying an electrical potential to arrays of cell cultures, while analyzing the reaction of the cell in terms of viability, morphological characteristics, and gene expression. However, numerous studies have failed to analyze the impact of the precise physicochemical characteristics of the materials employed for cell culture on the reported behaviors [23,24].

CPs are one of several types of materials that used for cell growth response to ES, and they are widely employed either solely or in conjunction with other polymers [16]. Numerous novel polymers are currently commercially available. Their derivatives primarily consist of aromatic rings or linear chains with alternating single and double bonds that permit the transit of electrons through conjugated orbitals [25]. These compounds combine the physical and processing qualities of polymers with the electrical and visual features of semiconductors. The architectures of the most popular CPs are used to create batteries that can be recharged, electrochromic devices, CPs, drug delivery systems, light-emitting diodes, sensors and biosensors, electrocatalysts, and corrosion protection devices [26]. CPs often have bandgap (Eg) values greater than 1.5 eV, making them insulating substances. The decrease in Eg values resulting from polymeric chain oxidation or reduction makes it possible for these substances to become conductors [27]. It has been suggested that CPs, such as poly (3,4-ethylene dioxythiophene) (PEDOT) and PPy, when doped with polysaccharides, such as hyaluronic acid and heparin, exhibit promising characteristics for use in the field of living tissue engineering. Specifically, CPs have the potential to serve as suitable materials for neural electrodes, owing to their ability to function as both biocompatible and biodegradable electroactive substances. The use of PPy in various oxidizing states is well documented in the literature. Bovine bone marrow stromal cells were the subject of an ES investigation by Schmidt et al., who employed PPy as a conductive substrate [28]. The quasi-reference electrode was used as the silver wire in the ES configuration, with a PPy electrode as the anode and a gold wire as the cathode. However, because CPs are hydrophobic, poor protein attachment and bioactivity result from the lack of an ideal cell-compatible surface [29].

**Figure 1:**
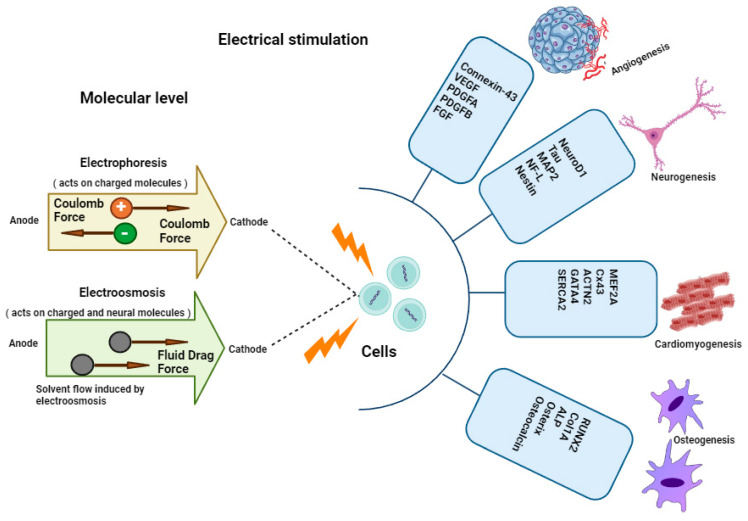
Multiple effects of ES at molecular and cellular levels.

ES has emerged as a unique method in the field of tissue engineering and is widely used in regenerative medicine (Figure 1). In tissue engineering, ES serves as a transformative tool, harnessing controlled electrical currents to direct and accelerate the growth and development of biological tissues. By modulating cellular behaviors, it fosters cell proliferation, orchestrates tissue organization, and steers cell differentiation, all of which are critical aspects of tissue regeneration. This technique finds particular significance in areas like neural tissue regeneration, bone repair, and wound healing, where it aids in the restoration of functional tissues and hastening the recovery process. The application of electrical stimulation, optimizing its parameters to maximize its impact and highlighting its indispensable role in advancing the field of tissue engineering, has been reported in recent studies. At the molecular level, it enhances the movement of both charged and uncharged biomolecules across biological membranes through mechanisms known as electroosmosis and electrophoresis. These two processes are collectively referred to as iontophoresis [22]. Electrical cues trigger a cascade of intracellular events. These signals can prompt the release of growth factors, such as vascular endothelial growth factor (VEGF) or nerve growth factor (NGF), which in turn stimulate cell proliferation and migration. Additionally, electrical stimulation can activate ion channels on cell membranes, leading to changes in intracellular calcium levels, which are crucial for various cellular processes. This intricate interplay of electrical signals within cells orchestrates a symphony of molecular events, ultimately fostering tissue growth, repair, and regeneration in the field of tissue engineering.

## 3. EAPs and Their Types

**Figure 2:**
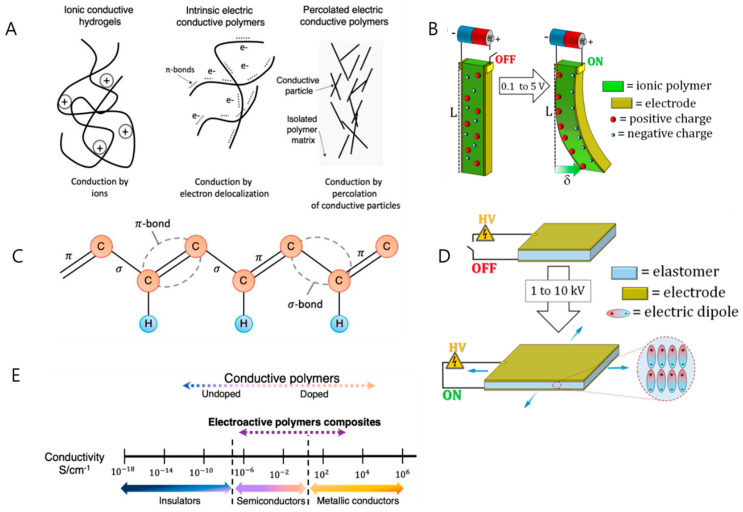
Types of EAPs. (**A**) different conduction mechanisms of EAPs [31]; (**B**) schematic representation of ionic electroactive polymer [32]; (**C**) electrically conductive polymers transfer electrons via intrinsic mechanism [31]; (**D**) schematic representation of two distinct families of electroactive polymeric actuators—electronic and ionic. Electroactive ionic polymer–metal composites, dielectric elastomers [32] (**E**) the conductivity spectrum of intrinsically conductive polymers and electroactive conductive material composites [31].

EAPs are a new class of organic materials with inherent conductivity that can be precisely controlled by altering their chemical and physical characteristics, depending on their intended use, such as molecular targeting, biosensors, or scaffolds for bioinstructive applications [20]. Their strongly conjugated electronic structure, which maintains charge mobility along the core of the polymer chain, is directly responsible for their superior conductive behavior. The fundamental benefits of these polymers stem from how simple they are to synthesize and how adaptable their chemical structure is, allowing materials to be modified as needed for different purposes [30]. Figure 2 presents various types of EAPs and their respective mechanisms.

EAPs can be divided into electronic and ionic groups based on various activation principles. Electronic EAPs (e-EAPs) actuate a polymer, such as a piezoelectric, electrostrictive, or ferroelectric material, to generate a contraction via the electrostatic attraction of two electrodes [30,33]. Based on ion movement and redistribution, when a voltage is applied, positively charged ions (cations) in the polymer move towards the negatively charged electrode (anode), while negatively charged ions (anions) move towards the positively charged electrode (cathode). This movement of ions forms an ion concentration gradient within the polymer, with a higher concentration of ions near the electrodes and a lower concentration in the middle. As ions gather near the metal electrodes, they form an electric double layer, which in turn causes the flow of water molecules through the polymer via electroosmosis. This water flow causes the polymer to become larger on one side and smaller on the other, making the ionic-polymer–metal composite (IPMC) turn or curve [31]. IPMCs have a quick reaction time because ions move quickly inside the polymer matrix. The ions usually move within the millisecond range. IPMCs are suitable for applications requiring quick movement, such as soft robotics and microfluidic devices, because they respond quickly. Ion migration and electroosmotic movement directly affect the way the IPMC bends and moves. The difference in ion concentration and water flow causes the polymer to grow and shrink at different locations along its length, causing it to bend. The angle of the bend can be adjusted by changing the voltage between the wires. As opposed to conventional EAPs, ionic EAPs (i-EAPs) actuate by dislodging the ions present in the polymer [30,33]. CPs, polymer–metal composites, and polyelectrolyte gels are some of the types of i-EAPs [32]. EAPs are most frequently used as actuators and sensors. Some EAPs have recently been developed for applications in tissue regeneration. For example, piezoelectric polymers operate without an external power source by providing electrical stimuli with a certain degree of control. CPs are biocompatible, exhibit strong optical properties, and have excellent conductivity-to-weight ratios [1].

Furthermore, CPs can exert precise control over the application of electrical stimulation. However, the operation of these devices requires an externally supplied electrical field. Moreover, CPs allow the manipulation of chemical, electrical, and physical properties to fulfill the specific needs of the biological elements in which they are employed. This review focuses on piezoelectric polymers, such as polyhydroxybutyrate, polyvinylidene fluoride (PVDF), and poly (L-lactic acid) (PLLA), as well as CPs, such as PPy, polyaniline (PANI), PEDOT, and polyelectrolyte gels. The objective of this review was to analyze their distinctive properties and applications in tissue regeneration. Each division can be further subdivided into numerous branches. Several EAPs have been developed that combine i-EAPs and e-EAPs to achieve specific benefits.

### 3.1. Ionic Electroactive Polymers

An i-EAP transfers electrical energy by passing ions via a polymer membrane. Thus, any polymer with carrier ions can be used for an i-EAP device’s outer layer [34]. According to their properties and material makeup, the polymers utilized in the polymer membrane of i-EAPs can be broadly classified as CPs, ionic polymer gels, electrochemical fluids, and i-EAPs. Each form of i-EAP is widely employed in many applications, including as sensors, actuators, and energy storage capacitors [1,2,3]. Despite their benefits, i-EAPs have limitations. One of the major drawbacks of IPMCs is that solvent evaporation from IPMC in a dry environment needs to be prevented, for instance, by replacing the metal electrode with a CP [15,31,34].

The two surfaces of the polymeric membrane have to be plated with conducting electrodes to generate or gather an electric signal across the thickness of the IPMC [35]. The two most common processes to attach metallic electrodes to ionic polymer membranes are ion exchange and chemical reduction. Noble metals, such as platinum or gold, are typically used to create electrodes, but alternative metals, including silver, copper, and palladium, have also been suggested. The manufacturing process involved the following steps.

- The surface of a polymer can be roughened by surface treatment, and preparing an ionomer surface increases the thickness of the surface area, facilitating metal salt reduction and penetration. This process maximizes the interface area between the polymer and the metallic layer. Ion exchange occurs within the polymer matrix, where metal ions are adsorbed onto the surface of the ionomer layer [35]
- The development stage, that is, secondary plating, involves growing an additional metal on top of the initial metal surface to lower the resistance of the electrodes. The deposited metal layers are then plated with additional metal layers.

The IPMC bending actuation has been demonstrated experimentally. An electrode strip device made of platinum (Pt) and a Nafion polymer membrane, known as the IPMC, was clamped to one end, and a voltage was supplied to cause it to bend. The main benefit of IPMCs is shown in this experiment, which is their large bending displacement (>360°) and quick reaction time of several seconds (>8 s). Ions that convey electrical energy are stored and moved through the polymer membrane of the IPMC [36]. Furthermore, the metal electrode serves as a conduit for transmitting electrical energy, facilitated by ions originating inside the polymer. To allow the enhancement of the performance characteristics, such as bending displacement, actuation force, and rapid reaction time, by reducing the surface resistance, the construction of this IPMC incorporates a combination of an ionized polymer membrane and a metal electrode. This review focuses on polymer membrane materials such as Nafion and other commonly used variants. We investigated many varieties of Nafion and other materials that are widely used in the field. Additionally, the manufacturing procedures of these polymer membrane materials were explored, with particular emphasis on the utilization of commercial Nafion film in its existing form, and with solution casting, hot pressing, and three-dimensional (3D) printing techniques. We primarily investigated the utilization of polymer and conventional metal electrodes for electrode fabrication [32].

### 3.2. Electronic Electroactive Polymers

In e-EAPs, an electric current is passed through the electrodes covered by a polymer membrane on either side to transfer the supplied energy to the polymer. The polymer membrane of the e-EAPs then develops an electric field. As a result, the Maxwell stress generated by the electric field inside the membrane results in the deformation of the polymer membrane’s shape [4]. In the initial inactive condition of the dielectric EAP, when triggered with a potential, the electric current applies Maxwell stress, causing the dielectric EAP to deform in thickness and area. Various polymer materials are utilized to create an electric field in e-EAP devices, and the type of material is a criterion for categorizing e-EAPs. e-EAPs can be divided into four categories: ferroelectric polymers, electroactive materials, dielectric elastomers, and electro-viscoelastic elastomers. e-EAPs that operate using electric current offer various benefits [5,6].

## 4. EAPs for Conductive Hydrogel Fabrications

i-EAPs can be fabricated using various materials. Based on these principles, ionic EAPs can be classified into several categories, including CPs, which can later be developed as IPMCs. However, if their properties are studied and understood, they can be combined to generate new i-EAPs. The following sections describe the synthesis techniques for various i-EAPs that fit into one or more of the aforementioned categories.

Researchers have developed unique approaches to exploit the potential biomedical applications of CPs. CPs are unique because of their simple synthesis methods and desirable electrical and optical properties, which are similar to those of metals and semiconductors. Polyacetylene has been shown to be conductive after oxidation by iodine vapor, which has attracted the interest of scientists. However, polyacetylene is unstable in air and challenging to synthesize. The most extensively investigated CPs are PEDOT, PANI, and PPy. Chemical or electrochemical synthesis of CPs is possible [37,38,39].

### 4.1. Polyaniline

PANI is affordable and has superior processability and strong environmental solidity [40,41]. It is a viable alternative to PPy and widely researched CPs. Therefore, many of PANI’s features are well known, including its wide range of structural configurations, affordable manufacturing, good processability, environmental stability, and ability to transfer charges via the “doping–de-doping” process [42]. Tests have shown that PANI is biocompatible in vitro and in vivo [43]. PANI and its variations have been shown by Bidez et al. to promote the adhesion and growth of H9c2 cardiac myoblasts [44]. Electrospinning may create nanofibers from PANI and gelatin mixtures to encourage H9c2 cell adhesion and proliferation [45]. These studies indicated the potential application of electroactive PANI in cardiac and nerve tissue regeneration. The nervous system is a close network of neurons that are excited by electric signals and transmit signals at an accelerated pace. Previously, various non-conductive scaffolds have been suggested to heal and regenerate damage to the central and peripheral nervous systems [45]. Since electrical inputs may activate neuronal function. Following the invention of CPs, scaffolds inspired by CPs have been used in neural regeneration. The positive regulation of neurite development and nerve regeneration by ES is now the subject of several ideas [46,47]. When built into scaffolds and activated by electrical signals, CPs, such as PPy and PANI, offer a novel technique to accommodate and promote the development and regeneration of nerve tissue devoid of growth factors [48].

### 4.2. Polypyrrole

Owing to its excellent biocompatibility, strong conductivity, environmental stability, simple manufacturing, and redox properties, PPy is one of the most studied CPs for applications in biomedicine [49]. According to several studies, PPy is compatible with various cells, including nerve cells, endothelial cells, cardiac cells, osteoblasts, and mesenchymal stem cells (MSCs) [7]. As the field of biomedical materials science advances, PPy has become an increasingly promising method for repairing and regenerating injured tissues. Neural cells exchange information via electric pulses because their membranes contain voltage-gated ion channels. Changes in the electrical potential close to these channels cause the channels to open. Electromagnetic stimulation of neuronal development, which simulates neuron–neuron communication, has been the subject of many studies. Because axons are lengthened through ES, PPy has been examined as a nerve conduit, both as a neural probe and a scaffold [50].

Yow et al. developed a collagen-based 3D fibrous scaffold containing PPy and demonstrated that human MSCs (hMSCs) cultured on PPy fibers display distinctive markers indicative of a neural lineage [51]. Additionally, they showed that external electric fields may affect cellular function and that prolonged stimulation could harm the culture framework [45]. It is thought that Schwann cell (SC) migration precedes and facilitates axonal healing in the peripheral nervous system [52]. Using electrical stimulation, Schmidt et al. [7] investigated SC behavior on PPy and how it affected neuronal regeneration. Their study showed that the average SC displacement increased in response to electrical stimulation, resulting in a net anodic migration effect.

Additionally, protein adsorption exhibits indirect impacts following film oxidation as a result of significantly impacting migration speed, but less impact on directionality. The electrical conductivity of PPy scaffolds gives them the potential to become a crucial component of cardiac and brain tissue engineering. A crucial aspect of the differentiation and functioning of neurons and myocytes is their response to electrical stimulation. PPy- or PANI-infused two-dimensional substrates or porous and fibrous scaffolds have recently been used to induce cardiac differentiation [53]. To allow electrical impulses to be transmitted at a typical speed in the right direction, cardiac cells must create appropriate intracellular networks and matrix topologies.

### 4.3. Poly(3,4-Ethylenedioxythiophene)

The development of an electronic ion-pump-based neurotransmitter delivery system in animal models has demonstrated the promising potential of PEDOT as a regenerative material [54]. Creating organic electrochemical transistors for biosensing is another application of PEDOT [55]. Creating a practical interface between electrically active tissues (such as the heart, skeletal muscle, or nervous system) and biomaterials is a significant area of research and development. Richardson et al. [56] investigated the effects of PEDOT on brain cells. They also developed microelectrodes, such as CP-live cell electrodes, by covering brain cells with PEDOT. Implantable electrodes made of PEDOT can be used to record neural activity or electrically stimulate nerves [57]. As an illustration, Green et al. created platinum electrodes covered with laminin peptides doped with PEDOT. The survival and growth of PC12 cells on the electrodes and the bioactivity of the peptides were subsequently studied. These findings indicated that large-peptide dopants produced softer PEDOT films. Longer neurites developed on laminin peptide-doped PEDOT films than on undoped films. The in situ chemical polymerization of PEDOT was investigated by Peramo et al. for the construction of acellular muscle tissue [58]. These studies demonstrate that in situ polymerization occurs throughout the tissue, changing it from a nonantigenic substrate to one with significant acellular properties, which is a crucial change for use in nerve healing and prostheses. Direct electrochemical polymerization of PEDOT creates CP matrices surrounding adherent cells when brain cells are seeded onto electrodes [59]. It was discovered that there is a close bond between the PEDOT surfaces and the neuronal membrane, which results in a unique polymer–electrode interface, while the surface is covered in frail filopodia and neurites [60]. When engineered to imitate cell membranes, the CPs created by Yu et al. showed several interesting properties, such as improved resistance to nonspecific enzyme–cell binding and targeted cell recognition when used in electrical communications with a very long duration. They found that the membrane mimics promoted neuronal cellular activity in response to a combination of pharmacological and electrical signals. In CPs, this effect was amplified, and neurite outgrowth was significantly affected [61].

### 4.4. Polythiophene

Thiophene derivatives provide several advantages, such as rapid polymerization, environmental stability, and high electrical conductivity/activity. Various polythiophene derivatives have been explored for use as tissue scaffolds. Chan et al. blended polythiophene phenylene (PThP) with bioresorbable poly (lactic-co-glycolic acid) and electrospun them into porous fiber mats. These mats were compatible with human dermal fibroblasts and epidermal melanocytes, indicating their suitability as tissue-engineering scaffolds. In addition, when PThP was functionalized with arginylglycylaspartic acid (RGD), cell proliferation was further enhanced. These findings suggest that PThP holds promise for developing tissue-engineering scaffolds [62].

In another study by Lee et al., carboxylic acid-functionalized PTh was investigated [63]. When PTh was modified with RGD, the adhesion of human dermal fibroblasts notably improved. This study underscores the potential utility of PTh derivatives in the design of scaffolds for skin tissue. Synthesized and electrochemically polymerized carboxylic-functionalized terthiophene, incorporating cuprous cyanide as one of the reagents [64]. They electrochemically polymerized films using a platinum (Pt) spiral wire as the counter electrode and an Ag/AgCl electrode as the reference electrode. In vitro culture experiments with human dermal fibroblasts revealed that the materials modified with arginylglycylaspartic acid supported the growth of fibroblasts and exhibited improved cell adhesion compared to unmodified controls. These findings suggest that bioactive conducting biomaterials have potential applications in various biomedical fields, including as bioelectrodes and tissue scaffolds [65].

### 4.5. Polyphenylene Sulfide

Polyphenylene sulfide (PPS) is a high-performance thermoplastic polymer known for its exceptional chemical resistance, high-temperature stability, and mechanical properties [66]. While PPS is not commonly used in tissue engineering, it may have limited applications in specific contexts. The primary challenge of using PPS in tissue engineering is its lack of biocompatibility and biodegradability. Tissue engineering typically involves materials that can provide a suitable environment for cell attachment, growth, and tissue regeneration, and eventually degrade into new tissue forms. As a non-biodegradable and chemically inert material, PPS does not inherently satisfy these requirements.

Researchers have explored the use of PPS as a component of composite materials or scaffolds in tissue engineering. In such cases, PPS may be blended with other biocompatible polymers or materials to create scaffolds with improved mechanical properties or thermal stability [67]. These composite materials may be used in specific tissue-engineering applications where long-term stability and resistance to harsh conditions are essential, such as bone tissue engineering or implantable devices. While PPS itself is not a typical choice for tissue engineering owing to its limited biocompatibility and biodegradability, it may find niche applications when incorporated into composite materials or in situations where its unique properties are required in combination with biocompatible materials [68]. Researchers continue to explore novel materials and approaches in tissue engineering to address specific challenges and requirements for different tissue regeneration applications. Table 1 summarize the applications of CPs in tissue regenerations along with its properties.

## 5. EAP–Metal Composites

Tissue engineering is a multidisciplinary field aimed at restoring or replacing damaged tissues and organs. It involves the use of scaffolds that provide a structural framework for cells to adhere to and grow, as well as bioactive factors that promote cell proliferation and tissue differentiation. Recently, there has been growing interest in developing EPMCs for use in tissue engineering. EPMCs are versatile materials with the potential to significantly enhance tissue-engineering applications [72]. These composites have unique properties, making them ideal candidates for various tissue-engineering applications. For example, the electroactive nature of these composites allows the generation of electrical signals in response to mechanical stimuli. The ability to convert mechanical energy into electrical signals is particularly advantageous in tissue engineering because it allows the stimulation of cells and tissues in a controlled manner to promote growth and regeneration. Moreover, EPMCs exhibit piezoelectric properties, which means that they generate electrical charges in response to mechanical stress. Piezoelectricity can further enhance the functionality of tissue-engineered constructs by providing a means of stimulating cell behavior and promoting tissue regeneration. The integration of EPMCs into tissue-engineered scaffolds offers several advantages. For example, these composites closely mimic the mechanical properties of natural tissues [1,72].

Because cells usually respond to mechanical signals in their surrounding microenvironment, the scaffold must offer an adequate environment for cell attachment and proliferation. Scientists are investigating the use of regenerative medicine and tissue engineering instead of utilizing transplants. Tissue engineering mostly makes use of scaffolds and targeted growth agents [73,74]. Recent developments in tissue engineering using EAPs and metal composites have the potential to bring cutting-edge technology to various biological domains [75]. Table 2 presents different CPs with metal composites and depicts their biological applications. IPMCs have numerous advantages over traditional intelligent materials, including high compliance, a low operating voltage, a light weight, and the ability to function in aquatic settings. These characteristics make IPMCs intriguing for various uses in biomedical, naval, robotic, and microelectromechanical system engineering. One of the essential uses of IPMCs is in biomedical devices that directly interact with human organs or tissues, such as artificial ventricular muscles, surgical instruments, and active scleral bands. Bands, rings, or shells are only a few possible configurations of IPMC materials for these purposes [76]. IPMCs are EAPs that have attracted attention as a practical actuator for several biomedical and industrial uses, including artificial muscles, robotic fish that swim underwater, human catheter systems, transducers, and micropumps [76]. Polymers (CPs) or polymers containing nanoparticles (NPs) with appropriate conductivity to substitute metallic electrodes on polymer membranes have recently been the primary focus of IPMC research. Examples include PEDOT Nafion with carbon nanotubes [77]. Since their benefits outweigh their downsides, i-EAPs are the subject of intensive study. However, IPMCs have certain disadvantages, including a short frequency response range and unstable performance under dry conditions. A notable disadvantage of IPMCs is their vulnerability to drying. IPMCs rely on the water within their polymer matrix to maintain their ionic conductivity and electroactive properties. In applications where maintaining a consistent moisture level is challenging, such as in certain dry environments or over extended operational durations, the performance of IPMCs can degrade significantly. The loss of water can lead to reduced ionic conductivity and impaired actuation capabilities [76].

Metal composites are important in biomedical engineering, particularly in tissue-engineering applications. Their unique combination of mechanical and electrical properties makes them well suited for mimicking the functionality of native tissues and promoting tissue growth and regeneration [74]. Because of their ability to imitate the electrical characteristics of native tissues, EAPMCs have emerged as viable materials for tissue-engineering applications. These composites have the potential to revolutionize tissue-engineering strategies by providing dynamic and responsive materials that can be controlled remotely. Integrating metals with EAPs in composite materials offers several advantages for tissue-engineering applications [78]. Firstly, the high flexibility of EAPMCs allows for easy shaping and manipulation into various scaffold designs, enabling the customization of scaffold constructs to suit specific tissue-engineering applications. Secondly, the low weight of these composites makes them ideal for implantable devices or structures within the body, thereby reducing the risk of mechanical strain or failure. Third, the electrical properties of EAPMCs enable them to interact with cells and tissues in a controlled manner [79]. These composites can electrically stimulate cells and promote their attachment to and growth on the scaffold.

Additionally, the electroactive properties of these composites can influence cellular behavior and promote tissue formation. EAPMCs have shown promise in various tissue-engineering applications. One specific application is in the development of active muscle tissue-engineering strategies. Electromechanically responsive polymers, such as piezoelectric polymers, have been utilized to develop active muscle tissue-engineering strategies that involve generating electrical responses under mechanical stimuli [80]. This capability allows the constructing of tissue constructs that can contract and expand in response to external stimuli, mimicking the functionality of native muscle tissue. Overall, EAPMCs have the potential to revolutionize tissue-engineering strategies by providing dynamic and responsive materials that mimic the electrical properties of native tissues.

By integrating metals with EAPs, tissue-engineering strategies can benefit from the unique electrical properties of composites. For example, in tissue engineering, IPMCs have been explored. These composites exhibit good mechanical and electrical actuation properties, making them ideal for applications requiring dynamic responses.

Microelectrodes, as a link between the tissue and an external device, can be used to achieve neural interface windows for biosignal recording and modulation, providing a promising platform for diagnosing or treating diseases such as Parkinson’s disease and hearing or visual disorders. The electrochemical resistance dramatically increases with the integration and shrinking of brain electrodes, resulting in more significant energy consumption and limiting their long-term usage [75]. Using noble metal nanocoatings (such as Pt, gold, or iridium) and carbon-based substances [81]. Researchers have attempted to modify the electrode surface to improve its performance. However, significant problems remain at neural interfaces. First, the stability of the electrode is weakened, and its long-term performance is constrained by in vivo metal or stiff-coating deterioration. After repeated stimulation with the electrode, the electroactivity of the material decreases. The coating materials and electrode locations also exhibit poor adhesion owing to mechanical delamination. Secondly, tissue reactions at the brain interface continue to pose significant challenges. A substantial non-conductive barrier is created between the metallic or stiff electrode and the soft tissue by chronic scarring and acute protein contamination, causing more signal loss [82,83].

### 5.1. Polypyrrole and Metal Composites

The chemical structure of PPy comprises repeating units of nitrogen-containing aromatic Py monomers. There is some debate regarding the mechanism of PPy polymerization. Nevertheless, it almost certainly includes intricate processes, including oxidation, deprotonation, and cross-linking [84]. P-doping, in which the polymer is oxidized and given a positive charge, is the doping method used for PPy [85]. According to one of the two dominant theories, PPy monomers are first oxidized to liberate a radical cation coupled with another cation to produce a pyrrole [84]. The chain keeps expanding as oxidation proceeds. The second theory proposes that cations combine with the neutral monomers. A dimer is created during oxidation and deprotonation, and the polymer chains continue to expand by repeating this procedure [86]. Metallic behavior eventually results in the delocalized electrons from the double bond in the PPy backbone becoming a conduction band.

PPy is a conducting polymer that has attracted considerable interest as an alternative to conventional scaffolding materials in tissue engineering. Adding metal NPs can improve the functionality of PPy-based scaffolds for tissue engineering. Gold, silver, and copper metal NPs have unique qualities that make them attractive candidates for tissue-engineering applications, such as outstanding biocompatibility and strong conductivity. Incorporating metal nanoparticles into PPy scaffolds can improve cell adhesion, proliferation, and differentiation owing to their conductive and catalytic properties [87]. Additionally, it has been demonstrated that PPy and metal composites can facilitate the proliferation of several cell types, including stem cells. This characteristic makes them appropriate for a broad spectrum of tissue-engineering applications. Researchers have also demonstrated the use of PPy–metal composites to create innovative materials that can respond to external stimuli, such as palladium (Pd)/PPy/rGO reduced graphene oxide) nanocomposites (NCs) [88].

In a recent study, PPy and Pd in a Pd/PPy/rGO NC produced surface functions and binding sites that improved the physicochemical properties of rGO for biomedical applications, employing rGO in two dimensions as a substrate for bone tissue engineering [89]. The rGO was non-covalently functionalized with PPy and Pd NPs to improve its biological features, such as biocompatibility, osteoproliferation, and bacterial infection prevention. Another study was conducted to create porous, biocompatible Ti substrates with a balanced biomechanical behavior (for the replacement of cortical bone tissue). Electropolymerization was used to coat these substrates with a composite CP made of PPy/silver NPs, improving their corrosion resistance and biocompatibility and increasing their antibacterial activity. PPy-based coatings that have been applied exhibit a cauliflower-like structure that is firmly attached to the porous surfaces [90].

### 5.2. Poly(3,4-Ethylenedioxythiophene) and Metal Composites

PEDOT, a CP, has been extensively investigated and applied in several disciplines, including tissue engineering. PEDOT exhibits unusual electroactive activity, making it appropriate for applications such as the control of medication distribution and neurological engineering. In the field of tissue engineering, PEDOT has demonstrated promising capabilities in nerve tissue engineering [91]. Advanced surface modifications, such as NH_2_-amine-functionalized treatment, interface chemistry, and carbon-sourced nanomaterials, have generated optimized functional materials. Research has improved PEDOT–metal interface adhesion and stability in brain electrodes [92]. Physical methods have increased substrate roughness, facilitating mechanical interlocking and surface area. The Pranti group used selective iodine etching on a gold (Au) electrode to create a textured surface that improved the mechanical interaction between PEDOT and the deposition of a protective poly(styrenesulfonate) (PSS) coating on a metal substrate. We aim to improve the stability of this system over time [93].

PEDOT:PSS has garnered significant interest in the fields of brain interfaces and biomedical applications, because of its excellent electrical properties and biocompatibility. Zeng described a CP hydrogel (CPH) composed of PEDOT and Wu copper (Cu), which was used as a sacrificial layer to successfully modify PSS on a platinum (Pt) substrate through electrochemical gelation. This CPH material has considerable potential for creating functionalized flexible electrodes, which suggests promising applications in flexible electronics and neurological implants [94]. The stability and delamination of PEDOT-coated metal electrodes are frequent problems, as they reduce the lifetime and performance of the device. Using precise platinum–iridium recording and stimulating brain electrodes, Bodart et al. successfully employed electropolymerization techniques to create PEDOT coatings. The authors demonstrated the electrochemical and mechanical durability of these coatings [95,96]. Three solvents, water, acetonitrile, and propylene carbonate, were used to electropolymerize tetrafluoroborate PEDOT. Ultrasonication was used to test the resistance of the coatings to deterioration, including soaking in a phosphate buffer solution, autoclave sterilization, and electrical pulsing [95].

### 5.3. Polyaniline and Metal Composition

PANI, a conducting polymer, was found to be promising and has been combined with several other components to expand its potential applications. One of the key advantages of PANI is its unique biocompatibility, which allows its use as a scaffold in tissue engineering. PANI can be made more processable in a composite form with EAPs, which act as stabilizers [97]. These included graphene, metal oxide, carbon, graphene oxide, carbon nanotubes, silicon dioxide, silica, and metal–organic frameworks.

Combining PANI with gold or silver NPs in tissue-engineering composites has several advantages [98]. The incorporation of PANI enhances the electrical conductivity of the composite material. This allows the transmission of electrical signals within the tissue, which is crucial for the proper functioning of electrically excitable tissues, such as cardiac and neural tissues [99]. Moreover, the tunable electrical properties of PANI allow precise control of the conductivity of the composite material. This is particularly important in tissue-engineering applications, where different tissues may require specific electrical conductivities for optimal functionality [100]. Second, gold and silver NPs offer unique properties that can benefit tissue-engineering applications [101]. These NPs have been shown to enhance the mechanical properties of tissues, improve cell adhesion and proliferation, and promote tissue regeneration [102]. Furthermore, gold and silver NPs possess antimicrobial properties that can help prevent infections in tissue-engineered constructs. Combining PANI with gold or silver NPs in tissue-engineering composites holds potential for various applications.

Hosseini et al. investigated the reaction mechanism of the Ag/PANI composite using numerous techniques [103]. A PANI–zinc–aluminum layered dihydroxide nanocomposite produced by free radical emulsion polymerization showed antibacterial activity [104]. Other solid structures, such as PANI-coated gold nanorods, have demonstrated antibacterial properties against *Escherichia coli* and *Staphylococcus aureus*. It has been reported that a PANI-based biosensor with Ag functionalization can be used to identify anticancer drugs [105]. PANI NCs containing microbicidal nanomaterials like zinc oxide and Ag nanocompounds have demonstrated synergistic antibacterial effects [106].

**Table 2:** Application of different electroactive polymer–metal composites.

Composite Name	Properties	Application	Key Responses	Ref.
PEDOT/Au or Pt	Biocompatible, highly conductive, flexible and stretchable, biodegradable	To accomplish neural interfaces’ frames for biosignal recording and modulation (treatment of Parkinson’s disease, hearing or visual disorders)	Enhance cell adhesion, improve electrical conductivity, enhance cell proliferation, and cell differentiation	[75]
PEDOT:PSS/Cu	Biocompatible, electrically conductive, antimicrobial, biodegradable	In flexible electronics and neurological implants	Enhance cell adhesion, control cell differentiation, support tissue regeneration	[94]
Pd/PPy/rGO	Biocompatible, electrically conductive, high surface area, biodegradable, catalytic	Improve the biological features osteoinductive infection prevention	Enhance cell adhesion, integrate with native tissues, stimulate tissue activity, support tissue regeneration	[89]
PPy/AgNPs	Biocompatible, electrically conductive, antimicrobial, facilitate cell signaling, biodegradable	Improved resistance to corrosion (replacement of cortical bone tissue) and biocompatibility and increased antibacterial activity	Enhance cell adhesion, improve electrical conductivity, and integrate with native tissues, prevent infections in tissues	[90]
PANI/Ag	Biocompatible, electrically conductive, antimicrobial, biodegradable, sensitive to changes in the environment	Neural tissue engineering	Enhance cell adhesion, improve electrical conductivity improve electrical conductivity, and integrate with native tissues, prevent infections in tissues	[106,107]
PANI/Pt Ni	Biocompatible, electrically conductive, biodegradable, catalytic, sensitive to changes in the environment	Enhanced electrocatalytic activity and other bioelectrochemical applications	Safe interaction, enhance cell adhesion, enhance cell viability, stimulate vascularization	[107,108]
PANI/Au Pd	Biocompatible, electrically conductive, catalytic, contrast agents of imaging modalities, biodegradable (depending upon the specific formula and design)	Improved antibacterial activity	Enhance cell proliferation and cell differentiation, enhance cell adhesion, enhance cell viability, stimulate vascularization	[108,109]

## 6. Synthesis of EAPMCs

One approach to designing and synthesizing EAPMCs involves the incorporation of an electroactive filler into an insulating biocompatible polymer matrix. The filler material can be selected based on its electroactive properties, such as the ability to generate electrical responses under mechanical stimuli. Commonly used electroactive fillers include piezoelectric polymers, ionic polymer gels, dielectric elastomers, and CP composites. Piezoelectric polymers are frequently employed when the objective is to produce electrical reactions in response to mechanical stimuli. Ionomeric polymer–metal composites, namely, electroactive polymer–metal composites, have emerged as promising materials for implementing active muscle tissue-engineering procedures. These composites consist of a thin polymer membrane with metal electrodes on both faces. They exhibit a significant and rapid bending response when a low voltage is applied between the electrodes. The design and synthesis of EAPMCs involve the careful selection of materials and fabrication techniques to achieve the desired properties.

The specific design and synthesis of EAPMCs require careful selection of the filler material and insulating biocompatible polymer matrix. The filler material should possess the desired electroactive properties, such as piezoelectricity or conductivity, to generate electrical responses under mechanical stimuli. An insulating biocompatible polymer matrix should provide structural support and ensure compatibility with the surrounding tissue. Furthermore, the fabrication techniques used in the synthesis process play a critical role in achieving optimal composite performance. Several advantages make EAPMCs promising materials for tissue-engineering applications. First, they exhibit considerable actuation strain, which indicates that they can undergo significant deformation in response to a small applied force or voltage. Second, they have a high response speed, which allows for fast and dynamic movements. Third, they have low density, making them lightweight and suitable for implantation or integration with biological tissues. Finally, the ease of molding and shaping provides a practical advantage for tissue-engineering applications. EAPMCs offer promising characteristics that make them suitable for tissue-engineering applications.

**Figure 3:**
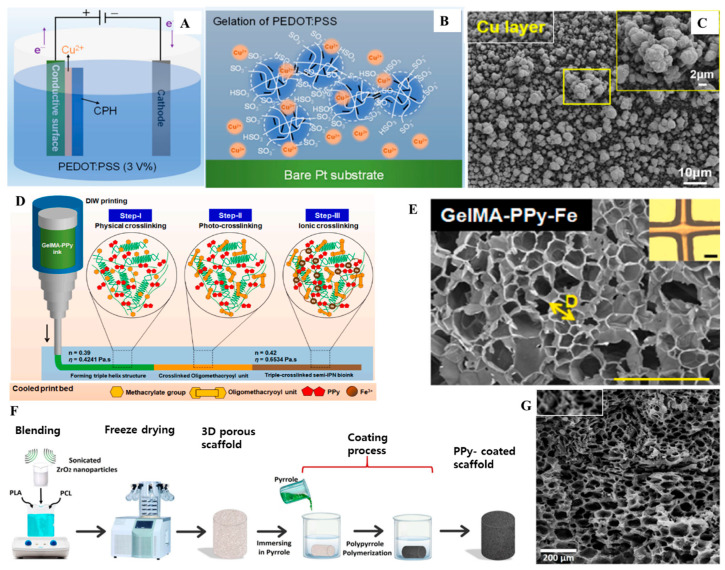
Fabrication of hydrogels based on EAPMC. (**A**,**B**) A conventional electrochemical cell for electrochemical gelation of aqueous PEDOT:PSS electrolyte. Under constant potential, Cu is entirely oxidized to Cu^2+^ ions [110]. (**C**) SEM image of Cu layer of PEDOT:PSS hydrogel (with modified Pt) [111]. (**D**) Schematic representation of fabrication of 3D-printed hydrogel with triple cross-linking mechanism. (**E**) SEM image of GelMA-PPy-Fe hydrogel, Yellow arrow indicates the pore diameter of corresponding images. (**F**,**G**) Schematic representation of fabrication of ZrO_2_ nanoparticle-incorporated 3D PCL/PLA scaffolds. The right side shows the SEM image of the PCL/PLA scaffold [111]. Based on [94].

The use of EAPMCs in tissue engineering shows great potential for the development of novel strategies to actively promote muscle tissue regeneration and functionality. These composites can generate an electrical response to mechanical stimuli, allowing dynamic and responsive movements that are crucial for mimicking the natural properties of muscle tissues. They can act as active actuators capable of bending and deforming in response to a low voltage applied between the metal electrodes. According to Zeng et al., a CPH constructed of PEDOT:PSS is effectively electrochemically gelated on a platinum (Pt) substrate using copper (Cu) as a sacrificial layer [94]. After drying in air, the as-prepared CPH electrode has a high surface area and excellent adhesion. Compared with bare Pt, the uniform flower-like structure allowed the electrode to attain a much lower square impedance of 10.09 cm^2^. (a reduction of 54.89%) at 1 kHz. The electrode has a high cathodic charge storage capacity of up to 82.36 mC cm^2^, which is about 27 and 16.18 times more than that of bare Pt and PEDOT:PSS coating, respectively. Moreover, after five cyclic voltammetry cycles in phosphate-buffered saline, no evident fluctuation in the charge storage capacity occurred, and a higher charge injection capacity was observed (Figure 3A–C).

Furthermore, incorporating EAPs into a biocompatible matrix allows for the creation of conductive biomaterials that can restore, maintain, or improve tissue function. Dutta et al. developed a stable and conductive bioink for direct ink writing-based 3D printing applications based on PPy-grafted gelatin methacryloyl (GelMA-PPy) with triple cross-linking (thermo-photo-ionically) [110]. During 3D printing, a triple-cross-linked hydrogel with a dynamic semi-inner penetrating polymer network demonstrated outstanding shear-thinning capabilities, increased form fidelity, and structural stability. The hydrogel ink in its original state exhibited flow behavior characterized as “plug-like non-Newtonian” flow when subjected to little disruption. The bioprinted GelMA-PPy-Fe hydrogel exhibited improved cytocompatibility (93%) with human bone marrow mesenchymal stem cells (hBMSCs) when subjected to microcurrent stimulation at 250 mV for 20 min daily (Figure 3D,E).

In a study, 3D porous bioscaffolds created using poly(ε-caprolactone) and poly(lactic acid) with varying concentrations of zirconia NPs (n-ZrO_2_) incorporated. The scaffolds were fabricated using a freeze-drying technique. Subsequently, a conductive layer was applied to the scaffold surface via PPy in situ polymerization. The resulting bioscaffolds demonstrated a desirable range of mechanical properties and electrical conductivities, meeting the criteria for a wide spectrum of tissue-engineering applications [111]. Applying a PPy coating to the scaffolds led to substantially increased hydrophilicity and an accelerated biodegradation rate. Additionally, there was a noticeable improvement in in vitro cell attachment, proliferation, and cell viability. These results suggested that the combined presence of n-ZrO_2_ and PPy within the system produced a significant synergistic effect, enhancing the overall characteristics of the fabricated 3D porous scaffolds. This makes them highly promising candidates for applications in tissue engineering and regenerative medicine (Figure 3F,G).

## 7. Biomedical Applications

### 7.1. Neural Tissue Regeneration

The capacity for axonal growth declines gradually during neural development. Synapses are connections between neurons that allow them to communicate information when in an electrically active state, integrating them into useful neural circuits [81]. The primary barriers to axon regeneration and operational reconstruction in the adult tissue milieu of the central nervous system are inhibitory factors and the poor innate capacity of neurons to regenerate.

Conductive graphitic-based materials can sustain neuronal survival, neurite development, and cellular electrical signaling. To create neural scaffolds, materials such as PPy [112] PEDOT [56] and PANI [113] are employed in constructing the scaffolds. Various bioactive surfaces have been created because of the flexibility of CP production. A conducting polymer that mimics the cell membrane showed the ability to electrically interface with neurons effectively and selectively. Preventing the development of immunogenic scars can facilitate neurite outgrowth while decreasing immune recognition.

Yow et al. created a collagen-based 3D fibrous scaffold containing PPy and demonstrated that human MSCs (hMSCs) cultured on PPy fibers displayed distinctive markers indicative of a neural lineage [51]. Additionally, they showed that external electric fields could affect cellular function and that prolonged stimulation could harm the culture system. According to the present consensus, migration of Schwann cells (SCs) in the peripheral nervous system precedes and facilitates axonal healing [52]. Schmidt et al. used ES to study the behavior of SCs on PPy and how it affected neuronal regeneration [7]. Their research showed that ES caused an upsurge in the average SC displacement, ultimately resulting in a net anodic migratory effect. Furthermore, protein adsorption had unintended consequences when the films were oxidized because ES significantly influenced the pace of migration, but not the directionality. Utilizing a conduit, SCs would move straight from the proximal to the distal end of the wound via ES. More neuronal regeneration, and perhaps more functional recovery, may occur as SC migration to the distal end increases. Huang et al. [52] showed that conductive PPy–chitosan coatings increase SC viability, adhesion, and spreading, with or without electrical stimuli. Interestingly, when compared to cells not subjected to electrical stimuli, nerve growth factor (NGF) and brain-derived neurotrophic factor synthesis were significantly increased by electrical stimuli administered through the PPy–chitosan composites [114]. These intriguing results demonstrate the value of PPy in nerve healing.

**Figure 4:**
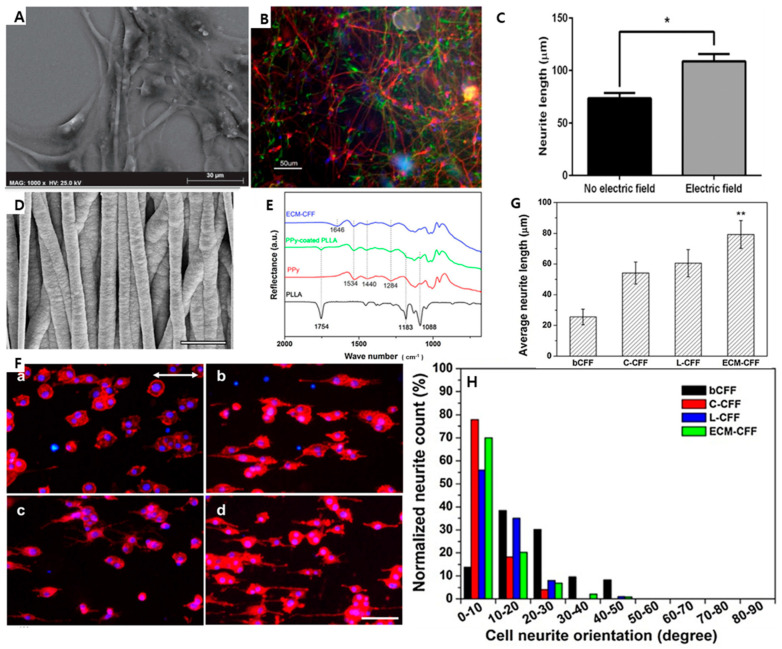
Different EAPMC-based hydrogel applications on neural tissue regeneration. (**A**) SEM images of PPy-coated PLLA fibers-film fabricated by electrochemical deposition [116]; (**B**) immunofluorescence image of cross-linked PEDOT:PSS with ES [116]; (**C**) an assessment of the pulsed DC electric field on the neurite length measured, * indicate sstatistically significant difference between values (*p* < 0.05) [116]; (**D**) electrochemically deposited PPy-coated PLLA fiber film [115]. Diameter 1.52 ± 0.25 μm; (**E**) ATR-FTIR spectra of PLLA, PPy, PPy-coated PLLA and ECM-CFF, respectively [106]; (**F**) immunofluorescent photographs of neurite proliferation after 48 h on (**a**) bCFF, (**b**) C-CFF, (**c**) L-CFF, and (**d**) ECM-CFF. The axis direction of the aligned fibers is shown by a double arrow. Scale bar in (**a**–**d**) is 100 m [115]; (**G**,**H**) typical neurite length and neurite alignment angle histograms from matching immunofluorescence pictures of PC12 cells cultivated for 48 h on bCFF, C-CFF, L-CFF, and ECM-CFF. ** Denotes statistical significance (*p* < 0.01) between ECM-CFF and other CFFs [115].

A previous study explored the cross-linking of PEDOT:PSS using an Au/Pd-layer hydrogel. Subsequently, ReNcellVM neural stem cells (NSCs) were introduced onto the laminin-coated surfaces and cultured. The culture medium contained EGF (20 ng/mL), FGF-2 (20 ng/mL), and B27 (20 g/mL) and cells were cultured for 4 days. Following this, the cells were subjected to a differentiation process for an additional 8 days, during which the aforementioned components were omitted. Simultaneously, the cells were electrically stimulated at 1 V with 10 ms pulses at a frequency of 100 Hz [115]. Scanning electron micrograph (SEM) images of electrically stimulated NSCs on cross-linked PEDOT are shown in Figure 4A. When using PSS substrates, electrical stimulation caused a change in the reorganization of the actin cytoskeleton, which appeared to be the source of the morphological changes in the cells described here, particularly stretching of the cells. Immunofluorescence analysis confirmed an increase in the neuronal population on the crosslinked PEDOT:PSS substrates upon exposure to ES, as shown in Figure 4B,C. Zhou et al. successfully synthesized a conductive fiber film (CFF) by electrospinning PLLA fibers and the subsequent electrochemical deposition of PPy NPs. The resulting CFF exhibited a core–sheath structure and demonstrated a conductivity of 10 S cm^−1^. This advancement holds promise for treating peripheral nerves [116]. Electrochemical deposition was used to create a PPy-coated PLLA fiber film. Figure 4D,E show the SEM images of the electrospun PPy-coated PLLA fibers. Attenuated total-reflection Fourier-transform infrared spectra of PPy, PLLA, PPy-coated PLLA, and ECM (extracellular matrix) CFFs (Figure 4F) show that nearly all of the cells were oriented along the fiber axis. More cells were observed with protruding neurites on C-CFF or LCFF than on bCFF, indicating that collagen- and laminin-coated fibers may stimulate PC12 cell differentiation and neurite growth. The average length of neurites was greater on ECM-CFFs (approximately 79 m) than on other CFFs (approximately 54 m on C-CFFs and 60 m on L-CFFs), demonstrating that ECM-CFFs greatly stimulated neurite growth. (Figure 4G). The orientation of neurites is shown in Figure 4H. The data revealed that a much higher proportion of neurites, 70% on ECM-CFFs and 77% on C-CFFs, expanded along the fiber axis compared to 14% on bCFF and 56% on L-CFFs. This finding strongly indicates that neurites on the ECM-CFF exhibit a notable tendency to extend along the fiber axis. The findings of this study demonstrated that the combination of ECM-CFF with NGF yielded substantial enhancements in both neurite development and direction [115].

### 7.2. Cardiac Tissue Engineering

The myocardium is an electrically active tissue that transmits and receives electrical impulses to regulate the heartbeat. In cardiac tissue engineering, electrically conductive biomaterials are particularly useful for repairing myocardial tissues after myocardial infarction owing to their effects on electrical signal propagation, improvements in cell–cell interactions, cardiomyocyte function for coordinating the beating of cardiomyocytes (CMs), and CM commitment.

Cardiovascular diseases are the leading cause of death globally [117]. Recent research has focused on generating functioning myocardial stents, cardiac patches, and heart valves as “spare parts” for cardiac repair. These “parts” may be used in a cardiac emergency. The electrical conductivity of PPy scaffolds may be essential for tissue engineering of the brain and heart. The ability to generate a response to ES is crucial for the development and function of neurons and muscle cells [118]. PPy and PANI have recently been infused into 2D substrates or porous fibrous scaffolds to enhance cardiac differentiation. Cardiac cells must establish proper intracellular networks and matrix topologies to ensure the regular bidirectional conduction of electrical impulses. The cardiac muscle contracts in response to electrical impulse waves, resulting in mechanical stretching of the heart.

Consequently, heart disease can develop owing to the disruption of the pathway that transmits electrical signals [119]. Recent studies have shown that a CP scaffold-based technique can facilitate heart tissue regeneration.

**Figure 5:**
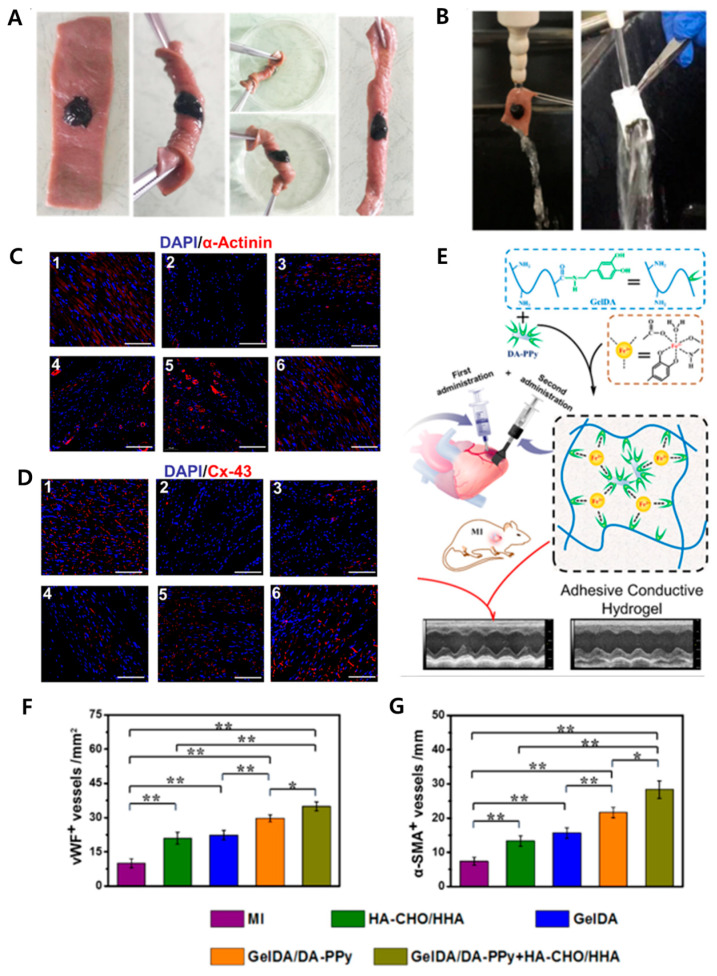
EAPs incorporated with metal-based hydrogel on cardiac tissue engineering. (**A**,**B**) GelDA/DA–PPy-0.6% hydrogel created in situ on porcine myocardium tissue exposes its robust adhesion; (**C**,**D**) immunofluorescent staining images of (**C**) α-actinin, (**D**) Cx-43. Scale bar = 100 μm; (**E**) schematic representation of the Fe^3+^-induced combined-internal–external therapy (CIET) of MI; (**F**,**G**) expression of the vWF and -SMA proteins determined the quantitative microvascular density and tiny arterioles in various groups (* *p* < 0.05, ** *p* < 0.01). Based on [121].

The myocardium has excellent conduction properties. PVDF is suitable for cardiovascular tissue engineering because of its unique properties of piezoelectric scaffolds. Lee et al. investigated the vitality and function of CMs and mouse embryonic stem cell-derived endothelial cells (mES-ECs) cultured on piezoelectric PVDF scaffolds [120]. Both mES-CMs and mES-ECs demonstrated good survival and adhesion to PVDF. Wu et al. investigated the development of Fe^3+^-induced ionic dexterity in the interaction between dopamine–gelatin conjugates and dopamine-functionalized PPy, specifically in the context of a self-adhesive conductive hydrogel patch (Figure 5A,B,E) [121]. The study employed immunofluorescence analysis to ascertain the cardiac structure within the infarcted area. To assess myocardial damage, the proteins actinin and Cx-43 were examined. Actinin is a crucial protein involved in the structure of the myocardium, while Cx-43 facilitates the transfer of electrical signals between myocardial cells [122]. When the myocardium is injured, the levels of both proteins are downregulated. In the myocardial infarction group, protein expression levels of actinin and Cx-43 were low. However, the expression of the two proteins increased in all hydrogel-treated groups, with the maximum expression levels of the two proteins occurring in the combination-treatment group (Figure 5C,D). Figure 5F,G show the quantitative microvascular density and arterioles in distinct groups based on the expression of vWF and SMA proteins.

### 7.3. Bone Tissue Engineering

The recent findings mentioned in the previous section showed that the composites rGO and PPy increase the mechanical strength of scaffolds, while Pd NPs eliminate bacterial biofilms [89]. Pd/PPy/rGO NC is a potential material for bone implantation because of the aforementioned intriguing properties. Because normal bone cells are not electrically excitable, the piezoelectric characteristics of bone tissues are the primary driver for the development of electroactive biomaterials for bone tissue engineering. To effectively create electroactive scaffolds for bone repair and regeneration under various disease conditions, it is imperative to have a comprehensive understanding of the bioelectrical characteristics inherent to the targeted bone tissue. These characteristics can be conceptualized as the interplay between inorganic and organic components beneath the biomechanical stimuli that arise from routine physical activities.

Consequently, several electrical phenomena, including dielectric, piezoelectric, pyroelectric, ferroelectric, electroosmotic, and streaming potentials, emerge as a result of this interaction. A thorough understanding of the bioelectrical properties of the target bone tissue is essential for designing electroactive scaffolds that promote bone regeneration and repair across a range of disease states. Composites using piezoelectric polymers have been demonstrated to promote osseointegration, osteogenesis, and ossification in in vivo investigations [110,123]. Liu et al. demonstrated that a multifunctional DOX-loaded GOMP (gelatin–oxidized dextran–montmorillonite strontium–polypyrrole) hydrogel, which has bone regeneration, photothermal therapy, and chemotherapeutic activities, shows great potential for the treatment of osteosarcoma [123]. The results of in vitro tumor therapy and in vivo antitumor recurrence experiments showed that the hydrogel containing DOX-loaded GOMP synergistically induced tumor cell death when used with chemotherapy and photothermal therapy.

**Figure 6:**
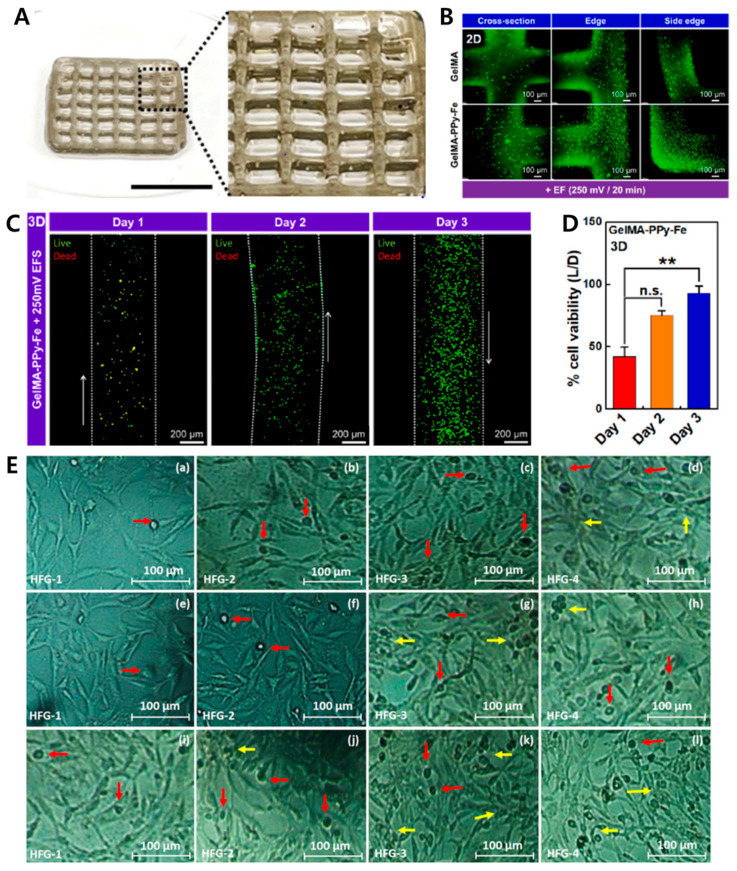
Bone tissue engineering applications. (**A**) 3D printed GelMA-PPy-Fe hydrogel. Scale bar: 10 mm [110]; (**B**) live/dead assay of hBMSCs after microcurrent stimulation treatment on 3D printed hydrogel [110]; (**C**,**D**) live/dead staining for 3D bioprinted hBMSCs after incubation and quantitative data. The arrow indicates the printing direction. Scale bar: 200 μm [110]; (**E**) cell morphology of MC3T3-E1 cell lines versus porous scaffolds at different time intervals (24, 48, 72 h) [124]. The red arrow shows the distribution of cells, and the yellow arrow shows the interconnected cells. GO—graphene oxide, HFG—halofuginone. ** *p* < 0.01. n.s. not significant.

Furthermore, doping the hydrogel with montmorillonite strontium enhanced the mechanical characteristics of the hydrogel and stimulated prospective bone regeneration. A recent study showed (Figure 6A) that fabricating a GelMA-PPy 3D-printed hydrogel is physiologically safe to use with ES to stimulate hBMSC growth [110]. FL (fluoroscopy) images with associated quantification of the live/dead staining of hBMSCs seeded onto the GelMA and GelMA-PPy-Fe hydrogels are shown in Figure 6B. Of note, even after microcurrent stimulation (EF-electrical field) activation, the number of cells increased by 15% in the presence of GelMA-PPy-Fe, indicating the biocompatibility of the conductive hydrogel (Figure 6C,D). Moreover, utilizing cellulose and a codispersed nanosystem (Fe_3_O_4_/GO) by free-radical polymerization, porous polymeric scaffolds were created and freeze-dried to study their structural, morphological, and mechanical properties. Khan et al. also performed swelling, biodegradation, and wetting analysis of the osteoblast cells, MC3T3-E1 [124]. After 72 h of culture, the morphology of the osteoblast cell lines was observed by microscopy (Figure 6Ee–Eh). Increases in the proliferation and adhesion of cells with a cylindrical shape were observed in response to increasing concentrations of Fe_3_O_4_, and GO was shown to have a favorable effect by providing more active sites owing to its oxygen-based functional groups. As shown in Figure 6, HFG-1 contained the fewest cells, whereas HFG-4 showed circular, concentrated cells and greater cell differentiation. Increased Fe_3_O_4_ induced pore size from HFG-1 to HFG-4 in all porous scaffolds. Polymeric NCs promote the mechanical strength of porous scaffolds, causing microcracks due to enhanced Fe_3_O_4_ levels, compressive strength, and Young’s modulus from HFG-1, and least for HFG-4. HFG-4 caused the greatest swelling and biodegradation, whereas HFG-1 caused the least. Fe_3_O_4_/GO and the polymeric matrix synergistically provided HFG-4 with the best antibacterial activity, cytocompatibility, cell proliferation, cell morphology, and adherence. Porous scaffolds with high antibacterial activity used in bone tissue engineering may cure damaged bones [124]. Table 3 provides a comprehensive overview of the many tissue regeneration applications using different EAPs, highlighting their respective advantages and limitations.

## 8. Advances and Challenges

Although EAPMCs show promise in tissue engineering, several challenges must be addressed for their successful implementation. The synthesis and design of EAP–metal composites for diverse tissue-engineering applications, such as bone, nerve, and cardiac tissue engineering, have recently been substantially improved. However, before they can be used extensively, these materials have several issues that must be resolved. The invention of innovative synthesis methods that enable the creation of materials with improved mechanical, electrical, and biological characteristics is one of the primary developments in EAP–metal composites [89]. For instance, the introduction of 3D printing, electrospinning, and other modern fabrication techniques has made it possible to produce composites with high porosity and well-defined microstructures, which can enhance their biocompatibility and ease cell integration. Furthermore, the addition of nanoparticles and other bioactive compounds to the composite matrix may boost the biological activity and effectiveness in tissue-engineering applications. The biological response of EAPMCs is paramount for their successful integration into tissue-engineering strategies. The interactions between EAPMCs and the surrounding biological environment play a pivotal role in determining the performance and efficacy of these materials. Biological reactions involving EAPMCs include how the body perceives and reacts to them. Implantable tissue-engineered materials must be biocompatible. Understanding how EAPMCs interact with surrounding tissues helps to discover biocompatibility concerns, such as inflammatory reactions or cytotoxicity, which may compromise the safety of implants or tissue-engineering constructs. Physical and biochemical stimuli from EAPMCs affect the surrounding cells. These interactions must be understood to achieve successful tissue engineering.

EAPMCs may exhibit cell growth or differentiation electrically, mechanically, or chemically. Understanding these pathways can help develop EAPMCs for tissue regeneration. One challenge is to ensure the biocompatibility of the composite materials. Incorporating metallic elements into a polymer matrix introduces the potential for toxicity or adverse reactions when in contact with living tissues. Researchers have focused on developing biocompatible polymer–metal composites to address this challenge by carefully selecting materials and optimizing their compositions. Surface modification techniques have also been explored to improve biocompatibility and reduce the risk of adverse reactions [67].

Tissue engineering is a complex challenge that researchers are actively addressing through several strategies, and material selection is critical. Researchers are opting for biocompatible polymers and metals that have a proven track record in medical applications. These materials must not induce toxic reactions or inflammation when in contact with living tissues. The release of intracellular ions triggered by the acidic environment of the lysosomal cellular compartment, where particles are frequently internalized, initiates a series of events linked to the intracellular toxicity induced by nanoparticles. This phenomenon is referred to as the “lysosome-enhanced Trojan horse effect”, as it highlights the paradoxical situation where the cellular machinery meant to break down foreign substances ends up causing their toxicity in the case of nanoparticles. Composition optimization is another avenue to fine-tune the ratios of polymers and metals within EAPMCs to achieve the desired mechanical and electrical properties while maintaining biocompatibility. This involves rigorous testing to ensure that the composite materials are stable and safe in biological environments. Surface modification techniques are also invaluable. Researchers are developing methods to modify the surface properties of EAPMCs to enhance their interaction with living cells. These modifications can include coatings that promote cell adhesion, minimize immune responses, and encourage tissue integration.

Moreover, the electrical conductivity of polymer–metal composites is a critical factor for their successful integration into tissue-engineering strategies [144]. To address this challenge, researchers have focused on enhancing the electrical conductivity of composites by incorporating highly conductive metals or carbon-based fillers into the polymer matrix. Additionally, optimizing the dispersion and connectivity of the metal particles within the polymer is crucial for ensuring efficient electrical conductivity throughout the composite material.

These challenges underscore the importance of a multidisciplinary strategy for creating EAPMCs for tissue engineering. Recent developments in bioelectronics have resulted in soft, tunable, electroactive organic materials that connect living organisms to electrical equipment. The application of these biomaterials in tissue engineering and regenerative medicine has gained attention because of their unique properties, including biocompatibility, controlled degradation, and the ability to create a porous network with good mechanical features 75. One key challenge in developing EAPMCs for tissue engineering is to ensure biocompatibility and reduce the risk of adverse reactions. Researchers have been actively working on incorporating biocompatible materials and optimizing their processing techniques to minimize the potential adverse reactions when using EAPMCs in tissue engineering. Furthermore, understanding the biological response of EAPMCs is essential for their successful integration into tissue-engineering strategies. The interaction between a composite material and the surrounding biological atmosphere can significantly influence its performance.

## 9. Conclusions and Future Perspectives

One potential future direction in this field is the development of bioactive EAPMCs that can promote cell adhesion and proliferation and tissue regeneration by incorporating bioactive molecules into the composite material or modifying its surface to enhance cell–material interactions. The incorporation of bioactive components with precise control over the release kinetics may enable tailored approaches for tissue regeneration and disease treatment. Future EAPMCs may feature surface modifications and nanostructured interfaces that enhance cell–material interactions. These modifications include nanotopography, biofunctional coatings, and the incorporation of cell-specific adhesion ligands. The goal is to create EAPMCs that closely mimic the natural extracellular matrix and provide an optimal environment for cell attachment, migration, and signaling. Another future direction is the integration of stimuli-responsive properties into electroactive polymer–metal composites, which can be achieved by incorporating responsive elements such as pH- or temperature-sensitive polymers into the composite, allowing for the tailored and controlled release of bioactive factors or drugs in response to specific physiological conditions. The development of EAPMCs with enhanced mechanical properties is another avenue for future tissue-engineering research.

Incorporating reinforcing fillers, such as NPs or fibers, into composite materials improves their strength and durability. Moreover, advances in tissue engineering have opened up opportunities for using EAPMCs in combination with other innovative techniques, such as 3D printing or bio-fabrication. These technologies allow the precise and customizable fabrication of complex tissue constructs with integrated electroactive properties. A multidisciplinary approach involving collaboration between materials scientists, biologists, engineers, and clinicians will be crucial for advancing the field of EAPMCs in tissue engineering. One potential direction for future research is the development of bioactive EAPMCs that actively promote cell adhesion and proliferation and tissue regeneration, which can be achieved by incorporating bioactive molecules into the composite material or modifying its surface to enhance cell–material interactions.

Furthermore, exploring novel synthesis methods and fabrication techniques for EAPMCs holds great promise for expanding their application in tissue engineering. For example, developing innovative manufacturing processes, such as electrospinning or 3D printing with conductive inks, can create complex and functional electroactive structures with precise control over their composition and architecture. Moreover, the characterization and understanding of the electrochemical behavior and enduring stability of EAPMCs in biological environments are crucial for their successful translation into clinical applications.

These materials possess unique properties that can contribute to the development of advanced tissue constructs with integrated electroactive functionalities. By incorporating electroactive fillers into biocompatible polymer matrices, these composites have the potential to enhance cell–material interactions and promote tissue regeneration. Future studies should focus on developing bioactive EAPMCs that promote cell adhesion and proliferation and tissue regeneration. Furthermore, integrating stimuli-responsive properties into these composites allows for the targeted and controlled release of bioactive factors in response to specific physiological conditions. Such advancements in tissue engineering using EAPMCs have the potential to revolutionize regenerative medicine by providing tailored and controlled approaches to promote tissue regeneration and improve patient outcomes.

In conclusion, the advent of EAPMCs has provided new opportunities for tissue engineering and regenerative medicine. These materials possess unique properties that can contribute to the development of advanced tissue constructs with integrated electroactive functionalities. Incorporating electroactive fillers into biocompatible polymer matrices has shown promise for enhancing cell–material interactions and promoting tissue regeneration.

However, further investigations are required to completely understand the electrochemical behavior and long-term stability of these composites in biological contexts. To successfully translate EAPMCs into therapeutic applications, it is essential to understand their electrochemical behavior and stability over time in biological settings. Future studies should examine the biocompatibility, toxicity, and compatibility of these composites with other biological materials and medicinal devices. Furthermore, efforts should be made to optimize the fabrication techniques and processing parameters of EAPMCs to ensure the consistency and reproducibility of their performance and properties.

## Figures and Tables

**Table 1:** Comparative analysis of CP utilized in various tissue regeneration.

Polymeric Material	Conductivity (S/cm)	Types of Doping	Properties	Applications	Ref.
Polypyrrole (PPy)	40–200	P	Amorphous, electrical conductivity high	Nerve, cardiac, and bone tissue engineering application	[18,69,70,71]
Polythiophene (PT)	10–100	P	Good electrical conductivity And optical property	Biosensing and neural tissue engineering	[18,72,73,74]
Polyaniline (PANI)	5,bulk films (up to 100 S/m)	N, P	Semi-flexible polymer requires simple doping/de-doping chemistry	Nerve and cardiac tissue engineering	[18,69,71]
Polythiophene (PT)	More than 100 S/cm	P	Semiconducting and optoelectronic properties, making it useful in organic electronics, flexibility	Neural tissue engineering	[59,61]
Poly phenylene sulfide	10–9 and 10–11 S/cm	P	High temperature resistance, good mechanical properties	Neural tissue engineering	[39,63]

(The number of outer electrons defines the type of doping. Elements with 3 valence electrons are used for P-type doping, and 5-valued elements for N-doping).

**Table 3:** Applications of EAPs in tissue engineering and regenerative medicine.

Tissue Regeneration Application	EAPs	Advantages	Limitations
Cartilage regeneration	Polyelectrolyte gel (PAMPS) [81,125]	Electroactive, adaptable, biocompatible, and encouraging cell differentiation	Lack of biodegradability and requirement for an additional power source
Neural regeneration	PPy [67] PANi [126] PEDOT [127,128] PVDF [129] PHB [130,131]	Good conductivity, biocompatibility, and stability, high specific surface area, easy processing, conducive to cell differentiation	Decreased electrical contact at the interface
Myocardial regeneration	PPy [132,133] PANi [134,135] PEDOT [136] Conductive polymer composites [137] PVDF [138]	Being electroactive, biocompatible, porous, fibrous, conducive to cell differentiation	Hydrophobicity, not biodegradable, needing an external power source
Bone tissue regeneration	PPy [139,140] PPY/PDLLA [69] Chitosan/PPy [141] PEDOT [68] PHB [130,142] Polyelectrolyte (PAMPS) [143]	Good biocompatibility and electroactivity, conducive to cell differentiation	Lack of biodegradability, hydrophobicity, needing an external power source

## References

[1] Ning C., Zhou Z., Tan G., Zhu Y., Mao C. (2018). Electroactive polymers for tissue regeneration: Developments and perspectives. Prog. Polym. Sci..

[2] Yang L., Zhang D., Zhang X., Tian A., Wang X. (2020). Models of displacement and blocking force of ionic-polymer metal composites based on actuation mechanism. Appl. Phys. A.

[3] Ariano P., Accardo D., Lombardi M., Bocchini S., Draghi L., De Nardo L., Fino P. (2015). Polymeric materials as artificial muscles: An overview. J. Appl. Biomater. Funct. Mater..

[4] Aziz M.R.F. (2020). A Novel Polypyrrole-Polyurethane Patch for Cardiac Electric Signal Restoration. Ph.D. Thesis.

[5] Rahman M.H., Werth H., Goldman A., Hida Y., Diesner C., Lane L., Menezes P.L. (2021). Recent progress on electroactive polymers: Synthesis, properties and applications. Ceramics.

[6] Yarali E., Baniasadi M., Zolfagharian A., Chavoshi M., Arefi F., Hossain M., Bastola A., Ansari M., Foyouzat A., Dabbagh A. (2022). Magneto-/electro-responsive polymers toward manufacturing, characterization, and biomedical/soft robotic applications. Appl. Mater. Today.

[7] Forciniti L., Ybarra J., Zaman M.H., Schmidt C.E. (2014). Schwann cell response on polypyrrole substrates upon electrical stimulation. Acta Biomater..

[8] Feng G.-H., Huang W.-L. (2016). Investigation on the mechanical and electrical behavior of a tuning fork-shaped ionic polymer metal composite actuator with a continuous water supply mechanism. Sensors.

[9] Liu X., Xing Y., Sun W., Zhang Z., Guan S., Li B. (2021). Investigation of the Dynamic Breakdown of a Dielectric Elastomer Actuator Under Cyclic Voltage Excitation. Front. Robot. AI.

[10] Zarei M., Samimi A., Khorram M., Abdi M.M., Golestaneh S.I. (2021). Fabrication and characterization of conductive polypyrrole/chitosan/collagen electrospun nanofiber scaffold for tissue engineering application. Int. J. Biol. Macromol..

[11] Amirouche F., Zhou Y., Johnson T. (2009). Current micropump technologies and their biomedical applications. Microsyst. Technol..

[12] He Z., Jiao S., Wang Z., Wang Y., Yang M., Zhang Y., Liu Y., Wu Y., Shang J., Chen Q. (2022). An Antifatigue Liquid Metal Composite Electrode Ionic Polymer-Metal Composite Artificial Muscle with Excellent Electromechanical Properties. ACS Appl. Mater. Interfaces.

[13] Wu S., Liu X., Yeung K.W., Liu C., Yang X. (2014). Biomimetic porous scaffolds for bone tissue engineering. Mater. Sci. Eng. R Rep..

[14] Marsudi M.A., Ariski R.T., Wibowo A., Cooper G., Barlian A., Rachmantyo R., Bartolo P.J. (2021). Conductive polymeric-based electroactive scaffolds for tissue engineering applications: Current progress and challenges from biomaterials and manufacturing perspectives. Int. J. Mol. Sci..

[15] Kaur G., Adhikari R., Cass P., Bown M., Gunatillake P. (2015). Electrically conductive polymers and composites for biomedical applications. RSC Adv..

[16] Yu R., Zhang H., Guo B. (2022). Conductive biomaterials as bioactive wound dressing for wound healing and skin tissue engineering. Nano-Micro Lett..

[17] Chen C., Bai X., Ding Y., Lee I.-S. (2019). Electrical stimulation as a novel tool for regulating cell behavior in tissue engineering. Biomater. Res..

[18] Ferrigno B., Bordett R., Duraisamy N., Moskow J., Arul M.R., Rudraiah S., Nukavarapu S.P., Vella A.T., Kumbar S.G. (2020). Bioactive polymeric materials and electrical stimulation strategies for musculoskeletal tissue repair and regeneration. Bioact. Mater..

[19] Schieker M., Seitz H., Drosse I., Seitz S., Mutschler W. (2006). Biomaterials as scaffold for bone tissue engineering. Eur. J. Trauma.

[20] Ghasemi-Mobarakeh L., Prabhakaran M.P., Morshed M., Nasr-Esfahani M.H., Baharvand H., Kiani S., Al-Deyab S.S., Ramakrishna S. (2011). Application of conductive polymers, scaffolds and electrical stimulation for nerve tissue engineering. J. Tissue Eng. Regen. Med..

[21] Huang X., Das R., Patel A., Duc Nguyen T. (2018). Physical stimulations for bone and cartilage regeneration. Regen. Eng. Transl. Med..

[22] Thrivikraman G., Boda S.K., Basu B. (2018). Unraveling the mechanistic effects of electric field stimulation towards directing stem cell fate and function: A tissue engineering perspective. Biomaterials.

[23] Talikowska M., Fu X., Lisak G. (2019). Application of conducting polymers to wound care and skin tissue engineering: A review. Biosens. Bioelectron..

[24] Ahadian S., Ostrovidov S., Hosseini V., Kaji H., Ramalingam M., Bae H., Khademhosseini A. (2013). Electrical stimulation as a biomimicry tool for regulating muscle cell behavior. Organogenesis.

[25] Schöbel L., Boccaccini A.R. (2023). A review of glycosaminoglycan-modified electrically conductive polymers for biomedical applications. Acta Biomater..

[26] Weng B., Diao J., Xu Q., Liu Y., Li C., Ding A., Chen J. (2015). Bio-interface of conducting polymer-based materials for neuroregeneration. Adv. Mater. Interfaces.

[27] Carlberg C., Chen X., Inganäs O. (1996). Ionic transport and electronic structure in poly (3, 4ethylenedioxythiophene). Solid State Ion..

[28] Schmidt C.E., Shastri V.R., Vacanti J.P., Langer R. (1997). Stimulation of neurite outgrowth using an electrically conducting polymer. Proc. Natl. Acad. Sci. USA.

[29] Gilmore K.J., Kita M., Han Y., Gelmi A., Higgins M.J., Moulton S.E., Clark G.M., Kapsa R., Wallace G.G. (2009). Skeletal muscle cell proliferation and differentiation on polypyrrole substrates doped with extracellular matrix components. Biomaterials.

[30] Simon D.T., Gabrielsson E.O., Tybrandt K., Berggren M. (2016). Organic bioelectronics: Bridging the signaling gap between biology and technology. Chem. Rev..

[31] Maksimkin A.V., Dayyoub T., Telyshev D.V., Gerasimenko A.Y. (2022). Electroactive polymer-based composites for artificial muscle-like actuators: A review. Nanomaterials.

[32] Palza H., Zapata P.A., Angulo-Pineda C. (2019). Electroactive smart polymers for biomedical applications. Materials.

[33] Almomani A.M. (2017). Study of Parameters Dominating Electromechanical and Sensing Response in Ionic Electroactive Polymer (IEAP) Transducers. Ph.D. Thesis.

[34] Le T.-H., Kim Y., Yoon H. (2017). Electrical and electrochemical properties of conducting polymers. Polymers.

[35] Olvera D., Monaghan M.G. (2021). Electroactive material-based biosensors for detection and drug delivery. Adv. Drug Deliv. Rev..

[36] Park S.W., Kim S.J., Park S.H., Lee J., Kim H., Kim M.K. (2022). Recent Progress in Development and Applications of Ionic Polymer–Metal Composite. Micromachines.

[37] Stříteský S., Marková A., Víteček J., Šafaříková E., Hrabal M., Kubáč L., Kubala L., Weiter M., Vala M. (2018). Printing inks of electroactive polymer PEDOT: PSS: The study of biocompatibility, stability, and electrical properties. J. Biomed. Mater. Res. Part A.

[38] Humpolíček P., Radaszkiewicz K.A., Capáková Z., Pacherník J., Bober P., Kašpárková V., Rejmontová P., Lehocký M., Ponížil P., Stejskal J. (2018). Polyaniline cryogels: Biocompatibility of novel conducting macroporous material. Sci. Rep..

[39] Mao J., Zhang Z. (2018). Polypyrrole as electrically conductive biomaterials: Synthesis, biofunctionalization, potential applications and challenges. Cutting-Edge Enabling Technologies for Regenerative Medicine.

[40] Xia Y., Lu X., Zhu H. (2013). Natural silk fibroin/polyaniline (core/shell) coaxial fiber: Fabrication and application for cell proliferation. Compos. Sci. Technol..

[41] Xia H., Tao X. (2011). In situ crystals as templates to fabricate rectangular shaped hollow polyaniline tubes and their application in drug release. J. Mater. Chem..

[42] Wu J., Tang Q., Li Q., Lin J. (2008). Self-assembly growth of oriented polyaniline arrays: A morphology and structure study. Polymer.

[43] Mattioli-Belmonte M., Giavaresi G., Biagini G., Virgili L., Giacomini M., Fini M., Giantomassi F., Natali D., Torricelli P., Giardino R. (2003). Tailoring biomaterial compatibility: In vivo tissue response versus in vitro cell behavior. Int. J. Artif. Organs.

[44] Guterman E., Cheng S., Palouian K., Bidez P., Lelkes P., Wei Y. (2002). Peptide-Modified Electroactive Polymers for Tissue Engineering Applications.

[45] Guo B., Sun Y., Finne-Wistrand A., Mustafa K., Albertsson A.-C. (2012). Electroactive porous tubular scaffolds with degradability and non-cytotoxicity for neural tissue regeneration. Acta Biomater..

[46] Patel N., Poo M.-M. (1982). Orientation of neurite growth by extracellular electric fields. J. Neurosci..

[47] Sisken B., Kanje M., Lundborg G., Herbst E., Kurtz W. (1989). Stimulation of rat sciatic nerve regeneration with pulsed electromagnetic fields. Brain Res..

[48] Li G.N., Hoffman-Kim D. (2008). Tissue-engineered platforms of axon guidance. Tissue Eng. Part B Rev..

[49] Zha Z., Deng Z., Li Y., Li C., Wang J., Wang S., Qu E., Dai Z. (2013). Biocompatible polypyrrole nanoparticles as a novel organic photoacoustic contrast agent for deep tissue imaging. Nanoscale.

[50] Fattahi P., Yang G., Kim G., Abidian M.R. (2014). A review of organic and inorganic biomaterials for neural interfaces. Adv. Mater..

[51] Yow S.-Z., Lim T.H., Yim E.K., Lim C.T., Leong K.W. (2011). A 3D electroactive polypyrrole-collagen fibrous scaffold for tissue engineering. Polymers.

[52] Huang J., Hu X., Lu L., Ye Z., Zhang Q., Luo Z. (2010). Electrical regulation of Schwann cells using conductive polypyrrole/chitosan polymers. J. Biomed. Mater. Res. Part A Off. J. Soc. Biomater. Jpn. Soc. Biomater. Aust. Soc. Biomater. Korean Soc. Biomater..

[53] Qazi T.H., Rai R., Dippold D., Roether J.E., Schubert D.W., Rosellini E., Barbani N., Boccaccini A.R. (2014). Development and characterization of novel electrically conductive PANI–PGS composites for cardiac tissue engineering applications. Acta Biomater..

[54] Simon D.T., Kurup S., Larsson K.C., Hori R., Tybrandt K., Goiny M., Jager E.W., Berggren M., Canlon B., Richter-Dahlfors A. (2009). Organic electronics for precise delivery of neurotransmitters to modulate mammalian sensory function. Nat. Mater..

[55] Mabeck J.T., Malliaras G.G. (2006). Chemical and biological sensors based on organic thin-film transistors. Anal. Bioanal. Chem..

[56] Richardson-Burns S.M., Hendricks J.L., Martin D.C. (2007). Electrochemical polymerization of conducting polymers in living neural tissue. J. Neural Eng..

[57] Asplund M., Thaning E., Lundberg J., Sandberg-Nordqvist A., Kostyszyn B., Inganäs O., von Holst H. (2009). Toxicity evaluation of PEDOT/biomolecular composites intended for neural communication electrodes. Biomed. Mater..

[58] Peramo A., Urbanchek M.G., Spanninga S.A., Povlich L.K., Cederna P., Martin D.C. (2008). In situ polymerization of a conductive polymer in acellular muscle tissue constructs. Tissue Eng. Part A.

[59] Richardson-Burns S.M., Hendricks J.L., Foster B., Povlich L.K., Kim D.-H., Martin D.C. (2007). Polymerization of the conducting polymer poly (3,4-ethylenedioxythiophene)(PEDOT) around living neural cells. Biomaterials.

[60] Zhu B., Luo S.-C., Zhao H., Lin H.-A., Sekine J., Nakao A., Chen C., Yamashita Y., Yu H.-H. (2014). Large enhancement in neurite outgrowth on a cell membrane-mimicking conducting polymer. Nat. Commun..

[61] Wang X., Li Y., Chen Y.M., Guo L., Wang Q., Xu C.Y. (2018). Preparation and properties of functional graphene/polyvinyl alcohol composite films. J. For. Eng..

[62] Baker C., Wagner K., Wagner P., Officer D.L., Mawad D. (2021). Biofunctional conducting polymers: Synthetic advances, challenges, and perspectives towards their use in implantable bioelectronic devices. Adv. Phys. X.

[63] Lee J.Y., Jeong E.-D., Ahn C.W., Lee J.-W. (2013). Bioactive conducting scaffolds: Active ester-functionalized polyterthiophene. Synth. Met..

[64] Ren W., Yang Y., Yang J., Duan H., Zhao G., Liu Y. (2021). Multifunctional and corrosion resistant poly (phenylene sulfide)/Ag composites for electromagnetic interference shielding. Chem. Eng. J..

[65] Kucińska-Lipka J., Gubanska I., Pokrywczynska M., Ciesliński H., Filipowicz N., Drewa T., Janik H. (2018). Polyurethane porous scaffolds (PPS) for soft tissue regenerative medicine applications. Polym. Bull..

[66] Tyagi N., Gambhir K., Kumar S., Gangenahalli G., Verma Y.K. (2021). Interplay of reactive oxygen species (ROS) and tissue engineering: A review on clinical aspects of ROS-responsive biomaterials. J. Mater. Sci..

[67] Gelmi A., Zhang J., Cieslar-Pobuda A., Ljunngren M.K., Los M.J., Rafat M., Jager E.W. (2015). Electroactive 3D materials for cardiac tissue engineering. Proceedings of the Electroactive Polymer Actuators and Devices (EAPAD).

[68] Shahini A., Yazdimamaghani M., Walker K.J., Eastman M.A., Hatami-Marbini H., Smith B.J., Ricci J.L., Madihally S.V., Vashaee D., Tayebi L. (2014). 3D conductive nanocomposite scaffold for bone tissue engineering. Int. J. Nanomed..

[69] Xu H., Holzwarth J.M., Yan Y., Xu P., Zheng H., Yin Y., Li S., Ma P.X. (2014). Conductive PPY/PDLLA conduit for peripheral nerve regeneration. Biomaterials.

[70] Aydın M., Aydın E.B., Sezgintürk M.K. (2019). A highly selective poly (thiophene)-graft-poly (methacrylamide) polymer modified ITO electrode for neuron specific enolase detection in human serum. Macromol. Biosci..

[71] Benny Mattam L., Bijoy A., Abraham Thadathil D., George L., Varghese A. (2022). Conducting Polymers: A Versatile Material for Biomedical Applications. ChemistrySelect.

[72] Wibowo A., Tajalla G.U., Marsudi M.A., Cooper G., Asri L.A., Liu F., Ardy H., Bartolo P.J. (2021). Green synthesis of silver nanoparticles using extract of Cilembu sweet potatoes (Ipomoea batatas L var. Rancing) as potential filler for 3D printed electroactive and anti-infection scaffolds. Molecules.

[73] Liu Z., Wan X., Wang Z.L., Li L. (2021). Electroactive biomaterials and systems for cell fate determination and tissue regeneration: Design and applications. Adv. Mater..

[74] Stewart E., Kobayashi N.R., Higgins M.J., Quigley A.F., Jamali S., Moulton S.E., Kapsa R.M., Wallace G.G., Crook J.M. (2015). Electrical stimulation using conductive polymer polypyrrole promotes differentiation of human neural stem cells: A biocompatible platform for translational neural tissue engineering. Tissue Eng. Part C Methods.

[75] Goding J.A., Gilmour A.D., Aregueta-Robles U.A., Hasan E.A., Green R.A. (2018). Living Bioelectronics: Strategies for Developing an Effective Long-Term Implant with Functional Neural Connections. Adv. Funct. Mater..

[76] Chen Z., Um T.I., Bart-Smith H. (2012). Bio-inspired robotic manta ray powered by ionic polymer–metal composite artificial muscles. Int. J. Smart Nano Mater..

[77] Neuhaus R., Zahiri N., Petrs J., Tahouni Y., Siegert J., Kolaric I., Dahy H., Bauernhansl T. (2020). Integrating ionic electroactive polymer actuators and sensors into adaptive building skins–potentials and limitations. Front. Built Environ..

[78] De Witte T.-M., Fratila-Apachitei L.E., Zadpoor A.A., Peppas N.A. (2018). Bone tissue engineering via growth factor delivery: From scaffolds to complex matrices. Regen. Biomater..

[79] Cheng H., Huang Y., Yue H., Fan Y. (2021). Electrical stimulation promotes stem cell neural differentiation in tissue engineering. Stem Cells Int..

[80] Wan X., Zhao Y., Li Z., Li L. (2022). Emerging Polymeric Electrospun Fibers: From Structural Diversity to Application in Flexible Bioelectronics and Tissue Engineering.

[81] Kostarelos K., Vincent M., Hebert C., Garrido J.A. (2017). Graphene in the design and engineering of next-generation neural interfaces. Adv. Mater..

[82] Chen N., Tian L., Patil A.C., Peng S., Yang I.H., Thakor N.V., Ramakrishna S. (2017). Neural interfaces engineered via micro-and nanostructured coatings. Nano Today.

[83] Gulino M., Kim D., Pané S., Santos S.D., Pêgo A.P. (2019). Tissue response to neural implants: The use of model systems toward new design solutions of implantable microelectrodes. Front. Neurosci..

[84] Tan Y., Ghandi K. (2013). Kinetics and mechanism of pyrrole chemical polymerization. Synth. Met..

[85] Guimard N., Gomez N., Schmidt C. (2007). Conducting polymers in biomedical applications. Prog. Polym. Sci..

[86] Sadki S., Schottland P., Brodie N., Sabouraud G. (2000). The mechanisms of pyrrole electropolymerization. Chem. Soc. Rev..

[87] Fattahi N., Ramazani A. (2023). Use of Nanomaterials in Bone Regeneration. Nanobiomater. Perspect. Med. Appl. Diagn. Treat. Dis..

[88] Sadat Z., Farrokhi-Hajiabad F., Lalebeigi F., Naderi N., Gorab M.G., Cohan R.A., Eivazzadeh-Keihan R., Maleki A. (2022). A comprehensive review on the applications of carbon-based nanostructures in wound healing: From antibacterial aspects to cell growth stimulation. Biomater. Sci..

[89] Murugesan B., Pandiyan N., Arumugam M., Sonamuthu J., Samayanan S., Yurong C., Juming Y., Mahalingam S. (2020). Fabrication of palladium nanoparticles anchored polypyrrole functionalized reduced graphene oxide nanocomposite for antibiofilm associated orthopedic tissue engineering. Appl. Surf. Sci..

[90] García-Cabezón C., Godinho V., Pérez-González C., Torres Y., Martín-Pedrosa F. (2023). Electropolymerized polypyrrole silver nanocomposite coatings on porous Ti substrates with enhanced corrosion and antibacterial behavior for biomedical applications. Mater. Today Chem..

[91] Shah S.A.A., Firlak M., Berrow S.R., Halcovitch N.R., Baldock S.J., Yousafzai B.M., Hathout R.M., Hardy J.G. (2018). Electrochemically enhanced drug delivery using polypyrrole films. Materials.

[92] Minh T.D., Lee B.-K. (2017). Effects of functionality and textural characteristics on the removal of Cd (II) by ammoniated and chlorinated nanoporous activated carbon. J. Mater. Cycles Waste Manag..

[93] Pranti A.S., Schander A., Bödecker A., Lang W. (2018). PEDOT: PSS coating on gold microelectrodes with excellent stability and high charge injection capacity for chronic neural interfaces. Sens. Actuators B Chem..

[94] Zeng Q., Wu T. (2022). Enhanced electrochemical performance of neural electrodes based on PEDOT: PSS hydrogel. J. Appl. Polym. Sci..

[95] Bodart C.M., Rossetti N., Hagler J.E., Chevreau P., Chhin D., Soavi F., Schougaard S.B., Amzica F., Cicoira F. (2019). Electropolymerized poly (3,4-ethylenedioxythiophene)(PEDOT) coatings for implantable deep-brain-stimulating microelectrodes. ACS Appl. Mater. Interfaces.

[96] Osmani R.A., Singh E., Kazi H., Bhosale R., Vaghela R., Patravale V. (2023). Conductive polymers and composite-based systems: A quantum leap in the drug delivery arena and therapeutics. Smart Polymeric Nano-Constructs in Drug Delivery.

[97] Veluru J.B., Satheesh K., Trivedi D., Ramakrishna M.V., Srinivasan N.T. (2007). Electrical properties of electrospun fibers of PANI-PMMA composites. J. Eng. Fibers Fabr..

[98] Abdi M.M., Md Tahir P., Liyana R., Javahershenas R. (2018). A surfactant directed microcrystalline cellulose/polyaniline composite with enhanced electrochemical properties. Molecules.

[99] Rahman S.U., Bilal S., ul Haq Ali Shah A. (2020). Synthesis and characterization of polyaniline-chitosan patches with enhanced stability in physiological conditions. Polymers.

[100] Nemati S., Kim S.-J., Shin Y.M., Shin H. (2019). Current progress in application of polymeric nanofibers to tissue engineering. Nano Converg..

[101] Ziai Y., Rinoldi C., Nakielski P., De Sio L., Pierini F. (2022). Smart plasmonic hydrogels based on gold and silver nanoparticles for biosensing application. Curr. Opin. Biomed. Eng..

[102] Huang Z.-H., Shi L., Zhu Q.-R., Zou J.-T., Chen T. (2010). Fabrication of Polyaniline/Silver Nanocomposite Under Gamma-ray Irradiation. Chin. J. Chem. Phys..

[103] Youssef A.M., Moustafa H.A., Barhoum A., Hakim A.E.F.A.A., Dufresne A. (2017). Evaluation of the Morphological, Electrical and Antibacterial Properties of Polyaniline Nanocomposite Based on Zn/Al-Layered Double Hydroxides. ChemistrySelect.

[104] Zahed F.M., Hatamluyi B., Lorestani F., Es’haghi Z. (2018). Silver nanoparticles decorated polyaniline nanocomposite based electrochemical sensor for the determination of anticancer drug 5-fluorouracil. J. Pharm. Biomed. Anal..

[105] Hou Y., Feng J., Wang Y., Li L. (2016). Enhanced antibacterial activity of Ag-doped ZnO/polyaniline nanocomposites. J. Mater. Sci. Mater. Electron..

[106] Blinova N.V., Stejskal J., Trchová M., Sapurina I., Ćirić-Marjanović G. (2009). The oxidation of aniline with silver nitrate to polyaniline–silver composites. Polymer.

[107] Babel V., Hiran B.L. (2021). A review on polyaniline composites: Synthesis, characterization, and applications. Polym. Compos..

[108] Yadav A., Pandey R., Liao T.-W., Zharinov V.S., Hu K.-J., Vernieres J., Palmer R.E., Lievens P., Grandjean D., Shacham-Diamand Y. (2020). A platinum–nickel bimetallic nanocluster ensemble-on-polyaniline nanofilm for enhanced electrocatalytic oxidation of dopamine. Nanoscale.

[109] Boomi P., Prabu H.G. (2013). Synthesis, characterization and antibacterial analysis of polyaniline/Au–Pd nanocomposite. Colloids Surf. A Physicochem. Eng. Asp..

[110] Dutta S.D., Ganguly K., Randhawa A., Patil T.V., Patel D.K., Lim K.-T. (2023). Electrically stimulated 3D bioprinting of gelatin-polypyrrole hydrogel with dynamic semi-IPN network induces osteogenesis via collective signaling and immunopolarization. Biomaterials.

[111] Jafari A., Mirzaei H., Shafiei M.A., Fakhri V., Yazdanbakhsh A., Pirouzfar V., Su C.-H., Ghaffarian Anbaran S.R., Khonakdar H.A. (2022). Conductive poly(ε-caprolactone)/polylactic acid scaffolds for tissue engineering applications: Synergy effect of zirconium nanoparticles and polypyrrole. Polym. Adv. Technol..

[112] Ateh D., Navsaria H., Vadgama P. (2006). Polypyrrole-based conducting polymers and interactions with biological tissues. J. R. Soc. Interface.

[113] Tahir Z.M., Alocilja E.C., Grooms D.L. (2005). Polyaniline synthesis and its biosensor application. Biosens. Bioelectron..

[114] Qi F., Wang Y., Ma T., Zhu S., Zeng W., Hu X., Liu Z., Huang J., Luo Z. (2013). Electrical regulation of olfactory ensheathing cells using conductive polypyrrole/chitosan polymers. Biomaterials.

[115] Zhou X., Yang A., Huang Z., Yin G., Pu X., Jin J. (2017). Enhancement of neurite adhesion, alignment and elongation on conductive polypyrrole-poly (lactide acid) fibers with cell-derived extracellular matrix. Colloids Surf. B Biointerfaces.

[116] Pires F., Ferreira Q., Rodrigues C.A., Morgado J., Ferreira F.C. (2015). Neural stem cell differentiation by electrical stimulation using a cross-linked PEDOT substrate: Expanding the use of biocompatible conjugated conductive polymers for neural tissue engineering. Biochim. Biophys. Acta (BBA)-Gen. Subj..

[117] Xu W., Topping M., Fletcher J. (2021). State of birth and cardiovascular disease mortality: Multilevel analyses of the National Longitudinal Mortality Study. SSM-Popul. Health.

[118] Bidez P.R., Kresh J.Y., Wei Y., Lelkes P.I. (2011). Enhanced cardiac differentiation of mouse embryonic stem cells by electrical stimulation. Stem Cell Engineering: Principles and Applications.

[119] Zimmermann W.-H., Didié M., Wasmeier G.H., Nixdorff U., Hess A., Melnychenko I., Boy O., Neuhuber W.L., Weyand M., Eschenhagen T. (2002). Cardiac grafting of engineered heart tissue in syngenic rats. Circulation.

[120] Hitscherich P., Wu S., Gordan R., Xie L.H., Arinzeh T., Lee E.J. (2016). The effect of PVDF-TrFE scaffolds on stem cell derived cardiovascular cells. Biotechnol. Bioeng..

[121] Wu T., Cui C., Huang Y., Liu Y., Fan C., Han X., Yang Y., Xu Z., Liu B., Fan G. (2019). Coadministration of an adhesive conductive hydrogel patch and an injectable hydrogel to treat myocardial infarction. ACS Appl. Mater. Interfaces.

[122] Hao T., Li J., Yao F., Dong D., Wang Y., Yang B., Wang C. (2017). Injectable fullerenol/alginate hydrogel for suppression of oxidative stress damage in brown adipose-derived stem cells and cardiac repair. ACS Nano.

[123] Liu X., Zhang Y., Wu H., Tang J., Zhou J., Zhao J., Wang S. (2023). A conductive gelatin methacrylamide hydrogel for synergistic therapy of osteosarcoma and potential bone regeneration. Int. J. Biol. Macromol..

[124] Khan M.U.A., Rizwan M., Razak S.I.A., Hassan A., Rasheed T., Bilal M. (2022). Electroactive polymeric nanocomposite BC-g-(Fe3O4/GO) materials for bone tissue engineering: In vitro evaluations. J. Biomater. Sci. Polym. Ed..

[125] Zhao W., Jin X., Cong Y., Liu Y., Fu J. (2013). Degradable natural polymer hydrogels for articular cartilage tissue engineering. J. Chem. Technol. Biotechnol..

[126] Wang L., Huang Q., Wang J.-Y. (2015). Nanostructured polyaniline coating on ITO glass promotes the neurite outgrowth of PC 12 cells by electrical stimulation. Langmuir.

[127] Collazos-Castro J.E., Polo J.L., Hernández-Labrado G.R., Padial-Cañete V., García-Rama C. (2010). Bioelectrochemical control of neural cell development on conducting polymers. Biomaterials.

[128] Sebaa M., Nguyen T.Y., Dhillon S., Garcia S., Liu H. (2015). The effects of poly (3,4-ethylenedioxythiophene) coating on magnesium degradation and cytocompatibility with human embryonic stem cells for potential neural applications. J. Biomed. Mater. Res. Part A.

[129] Royo-Gascon N., Wininger M., Scheinbeim J.I., Firestein B.L., Craelius W. (2013). Piezoelectric substrates promote neurite growth in rat spinal cord neurons. Ann. Biomed. Eng..

[130] Chang H., Wang Z., Luo H., Xu M., Ren X., Zheng G., Wu B., Zhang X., Lu X., Chen F. (2014). Poly (3-hydroxybutyrate-co-3-hydroxyhexanoate)-based scaffolds for tissue engineering. Braz. J. Med. Biol. Res..

[131] Khorasani M., Mirmohammadi S., Irani S. (2011). Polyhydroxybutyrate (PHB) scaffolds as a model for nerve tissue engineering application: Fabrication and in vitro assay. Int. J. Polym. Mater..

[132] Kai D., Prabhakaran M.P., Jin G., Ramakrishna S. (2011). Polypyrrole-contained electrospun conductive nanofibrous membranes for cardiac tissue engineering. J. Biomed. Mater. Res. Part A.

[133] Bidez P., Kresh J., Wei Y., Lelkes P. (2011). Stem Cell Engineering: Principles and Applications.

[134] Borriello A., Guarino V., Schiavo L., Alvarez-Perez M., Ambrosio L. (2011). Optimizing PANi doped electroactive substrates as patches for the regeneration of cardiac muscle. J. Mater. Sci. Mater. Med..

[135] Zhang J., Qiu K., Sun B., Fang J., Zhang K., Hany E.-H., Al-Deyab S.S., Mo X. (2014). The aligned core–sheath nanofibers with electrical conductivity for neural tissue engineering. J. Mater. Chem. B.

[136] Karagkiozaki V., Karagiannidis P., Gioti M., Kavatzikidou P., Georgiou D., Georgaraki E., Logothetidis S. (2013). Bioelectronics meets nanomedicine for cardiovascular implants: PEDOT-based nanocoatings for tissue regeneration. Biochim. Biophys. Acta (BBA)-Gen. Subj..

[137] Martins A.M., Eng G., Caridade S.G., Mano J.F., Reis R.L., Vunjak-Novakovic G. (2014). Electrically conductive chitosan/carbon scaffolds for cardiac tissue engineering. Biomacromolecules.

[138] Hwang G.T., Park H., Lee J.H., Oh S., Park K.I., Byun M., Park H., Ahn G., Jeong C.K., No K. (2014). Self-powered cardiac pacemaker enabled by flexible single crystalline PMN-PT piezoelectric energy harvester. Adv. Mater..

[139] Alvarez-Mejia L., Morales J., Cruz G.J., Olayo M.-G., Olayo R., Díaz-Ruíz A., Ríos C., Mondragón-Lozano R., Sánchez-Torres S., Morales-Guadarrama A. (2015). Functional recovery in spinal cord injured rats using polypyrrole/iodine implants and treadmill training. J. Mater. Sci. Mater. Med..

[140] Meng S., Rouabhia M., Zhang Z. (2013). Electrical stimulation modulates osteoblast proliferation and bone protein production through heparin-bioactivated conductive scaffolds. Bioelectromagnetics.

[141] Sajesh K., Jayakumar R., Nair S.V., Chennazhi K. (2013). Biocompatible conducting chitosan/polypyrrole–alginate composite scaffold for bone tissue engineering. Int. J. Biol. Macromol..

[142] Hayati A.N., Rezaie H., Hosseinalipour S. (2011). Preparation of poly (3-hydroxybutyrate)/nano-hydroxyapatite composite scaffolds for bone tissue engineering. Mater. Lett..

[143] Coimbra P., Ferreira P., De Sousa H., Batista P., Rodrigues M., Correia I., Gil M. (2011). Preparation and chemical and biological characterization of a pectin/chitosan polyelectrolyte complex scaffold for possible bone tissue engineering applications. Int. J. Biol. Macromol..

[144] Li X., Zheng F., Wang X., Geng X., Zhao S., Liu H., Dou D., Leng Y., Wang L., Fan Y. (2023). 1Biomaterial inks for extrusion-based 3D bioprinting: Property, classification, modification, and selection. Int. J. Bioprint..

